# Delivery of Biomimetic Liposomes via Meningeal Lymphatic Vessels Route for Targeted Therapy of Parkinson’s Disease

**DOI:** 10.34133/research.0030

**Published:** 2023-01-30

**Authors:** Jing Liu, Duyang Gao, Dehong Hu, Siyi Lan, Yu Liu, Hairong Zheng, Zhen Yuan, Zonghai Sheng

**Affiliations:** ^1^ Faculty of Health Sciences, Centre for Cognitive and Brian Sciences, University of Macau, Macau SAR 999078, P. R. China.; ^2^ Paul C. Lauterbur Research Center for Biomedical Imaging, Key Laboratory for Magnetic Resonance and Multimodality Imaging of Guangdong Province, Shenzhen Key Laboratory of Ultrasound Imaging and Therapy, CAS Key Laboratory of Health Informatics, Institute of Biomedical and Health Engineering, Shenzhen Institute of Advanced Technology, Chinese Academy of Sciences, Shenzhen 518055, P. R. China.

## Abstract

Targeted therapy of Parkinson’s disease is an important challenge because of the blood–brain barrier limitation. Here, we propose a natural killer cell membrane biomimetic nanocomplex (named BLIPO-CUR) delivered via the meningeal lymphatic vessel (MLV) route to further the therapeutic efficacy of Parkinson’s disease. The membrane incorporation enables BLIPO-CUR to target the damaged neurons, thus improving their therapeutic efficacy through clearing reactive oxygen species, suppressing the aggregation of α-synuclein, and inhibiting the spread of excess α-synuclein species. Compared with the conventional intravenous injection, this MLV administration can enhance the delivered efficiency of curcumin into the brain by ~20 folds. The MLV route administration of BLIPO-CUR enhances the treatment efficacy of Parkinson’s disease in mouse models by improving their movement disorders and reversing neuron death. Our findings highlight the great potential of MLV route administration used as targeted delivery of drugs to the brain, holding a great promise for neurodegenerative disease therapy.

## Introduction

Parkinson's disease (PD), a neurodegenerative encephalopathy, seriously endangers human health [1,2]. The clinical treatments for PD mainly contain chemical drugs (e.g., dopaminergic-type drugs, anticholinergics, or glutamate antagonist) through oral administration or intravenous administration [3–7]. However, the blood–brain barrier (BBB) greatly decreases the efficiency of drugs entering the brain and reduces their therapeutic effect [8,9]. To overcome this issue, nasal administration of drugs has been used as an alternative approach to bypassing the BBB [10–14]. Despite the fact that great achievements have been made, the enzymatic degradation in the nasal cavity and the mucus endothelium barriers may decrease the activity of drugs and reduce their absorption, respectively [12]. Therefore, it is crucial to explore effective drug delivery routes for improving the therapeutic efficacy of PD.

Meningeal lymphatic vessels (MLVs) have been considered an important drainage system to maintain intracranial fluid balance and immune monitoring [13–19]. Very recently, nanomedicine has been delivered into the brain via MLV route for glioma therapy, confirming the feasibility of this delivery route [20,21]. However, the abundant immune cells in the MLV route can endocytose the nanomedicine, thereby dramatically reducing the drug transport efficiency. In addition, the low targeting ability of these nanomedicines reduces their therapeutic efficacy with increasing side effects.

**Fig. 1.**
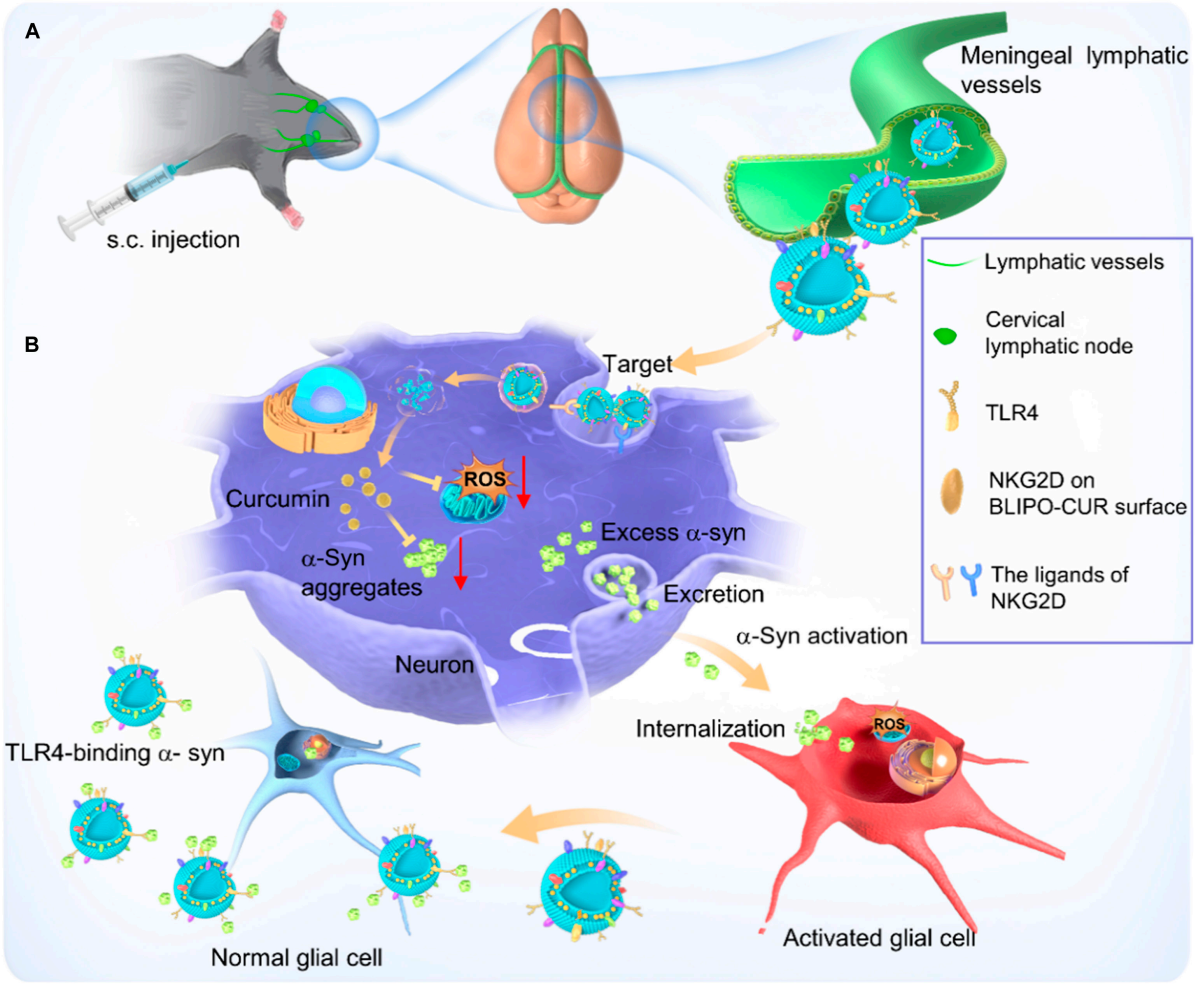
Schematic illustration of BLIPO-CUR delivered through MLVs and enhanced therapy for PD. (A) Subcutaneous (s.c.) administration of BLIPO-CUR at the neck of mice. (B) After crossing the MLVs, BLIPO-CUR could target the broken dopaminergic neurons through the integrations of the NKG2D receptor and its ligands. After the internalization of BLIPO-CUR, curcumin could clear the ROS and inhibit the α-syn to aggregate. The extracellular BLIPO-CUR could segregate the excess α-syn species among neurons and glial cells by TLR4 binding with α-syn.

To this regard, we reported an MLV delivery strategy for targeted therapy of PD using natural killer (NK) cell membrane-camouflaged curcumin liposomes (BLIPO-CUR) (Fig. 1). The biomimetic liposomes spread into the MLVs from cervical lymph nodes after subcutaneous injection in the neck site, escaping from the phagocytosis of macrophages and specifically recognizing the damaged neurons. Besides that, BLIPO-CUR could efficiently clear reactive oxygen species (ROS) and absorb α-synuclein (α-syn) species owning to the expression of Toll-like receptor 4 (TLR4) on NK cell surface. What is more, BLIPO-CUR could inhibit the spread of α-syn between neurons and glial cells as well as astrocytes and microglials, leading to enhanced PD therapeutic efficiency of curcumin. Our results hold great promise for the delivery of biomimetic liposome nanomedicines for efficient therapy of neurodegenerative encephalopathy.

## Results

### Preparation and characterization of BLIPO-CUR

**Fig. 2.**
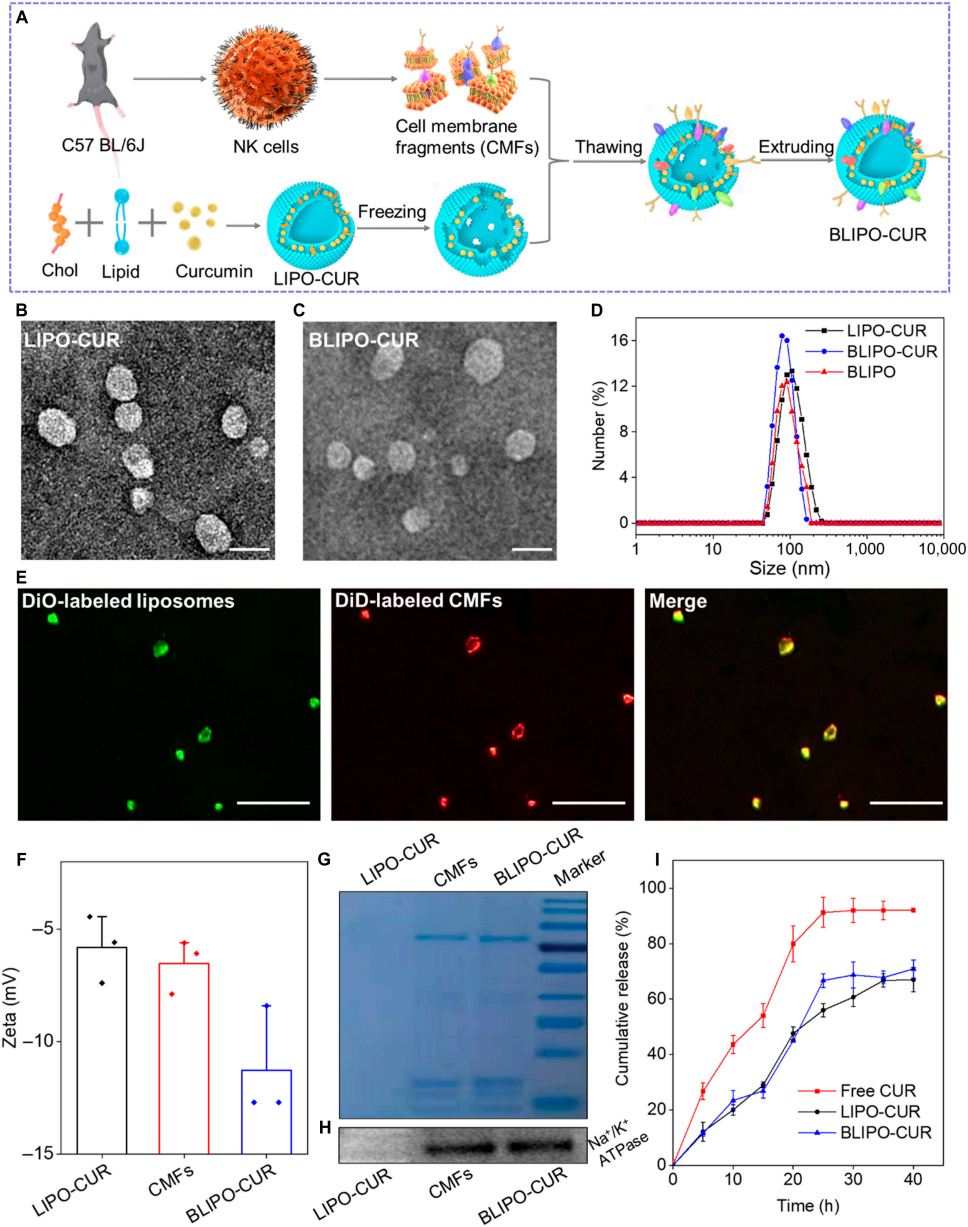
Preparation and characterization of BLIPO-CUR. (A) Schematic illustration of the preparation of BLIPO-CUR. (B and C) Transmission electron microscopy images of LIPO-CUR and BLIPO-CUR. Scale bars, 50 nm. (D) The size of LIPO-CUR, BLIPO-CUR, and BLIPO in PBS buffer (pH 7.4). (E) Fluorescence images of the mixture of liposomes (DiO, green) and CMFs (DiD, red) after being subjected to freeze–thaw process. Scale bars, 2.5 μm. (F) Zeta potential of LIPO-CUR, CMFs, and BLIPO-CUR in double-distilled H_2_O. (G) The image of sodium dodecyl sulfate-polyacrylamide gel electrophoresis within LIPO-CUR, BLIPO-CUR, and CMFs. (H) Western blot results of LIPO-CUR, BLIPO-CUR, and CMFs. (I) Accumulated drug release of free curcumin, LIPO-CUR, and BLIPO-CUR as a function of time in PBS (pH 7.4) containing 20% ethanol. ATPase, adenosine triphosphatase.

BLIPO-CUR was prepared by the repeated freezing–thawing of the mixtures of curcumin-loaded liposomes (LIPO-CUR) and NK cell membrane fragments (CMFs) derived from homologous murine NK cells (Fig. 2A and Fig. S1). After these physical treatments, the CMFs were embedded into the phospholipid bilayer of liposome shells. The spherical morphologies of LIPO-CUR and BLIPO-CUR were revealed through transmission electron microscopy images (Fig. 2B and C). In addition, the size of the LIPO-CUR, BLIPO-CUR, and BLIPO were measured to be around 100, 82, and 83 nm, respectively (Fig. 2D). The slightly decreased size of BLIPO-CUR was attributed to the successive extrusion for incorporation of CMFs into the phospholipid bilayer, and the smaller size facilitated the infiltration of nanoparticles into lymphatic vessels [22]. Fluorescence microscope imaging of BLIPO-CUR aggregates showed a good colocalization of the fluorescent channels in DiO-labeled lipids (green) and DiD-labeled NK CMFs (red) (Fig. 2E), confirming that the NK CMFs were anchored into the LIPO-CUR after freezing–thawing procedure. In contrast, the mixture of LIPO-CUR and NK CMFs without freezing and thawing treatments showed independent green and red dotted signals (Fig. S2). In addition, the zeta potential of LIPO-CUR was decreased from around −5.8 mV to around −11.3 mV after the incorporation of CMFs, confirming that the NK CMFs were embedded into the liposomes (Fig. 2F). Furthermore, surface proteins on CMFs and BLIPO-CUR were identified using sodium dodecyl sulfate-polyacrylamide gel electrophoresis and Western blotting. The similar protein profiles of NK CMFs and BLIPO-CUR demonstrated that the membrane proteins of the NK CMFs were preserved after incorporation into the liposomes (Fig. 2G and H).

The optical properties of curcumin were characterized after loading into the biomimetic liposomes. The absorption spectrum of BLIPO-CUR showed similar features as free curcumin (Fig. S3A), while the fluorescence emission was blue-shifted by ~24 nm compared to free curcumin, because of the fact that the fluorescence of curcumin was very sensitive to the polarity of the medium [23]. The encapsulation efficiency (EE) and loading efficiency (LE) of BLIPO-CUR were measured to be 66.7 ± 2.1% and 12.6 ± 0.3%, respectively (Table S1 and Fig. S3B and C), which were similar to previous reports [7,24]. Drug release behavior is a key factor to affect therapeutic efficiency. To investigate the release of curcumin from BLIPO-CUR, we measured the concentrations of curcumin after dialysis in phosphate-buffered saline (PBS) containing 20% ethanol at different time points. As shown in Fig. 2I, the cumulative release efficiency of free curcumin reached around 80% after dialysis for 20 h. In contrast, the release efficiency of BLIPO-CUR and LIPO-CUR was about 43%, reducing the release rate of about 2-folds. It indicated that the BLIPO-CUR and LIPO-CUR both exhibited a sustained-release effect. In addition, the hydrodynamic sizes of BLIPO-CUR in different media almost did not change during a 6-day observation (Figs. S3D and S4), indicating good stability. Thus, the encapsulation of curcumin into biomimetic liposomes could not only improve its water solubility and stability but also enable sustained-release behavior.

### Active targeting abilities of BLIPO-CUR in vitro

**Fig. 3.**
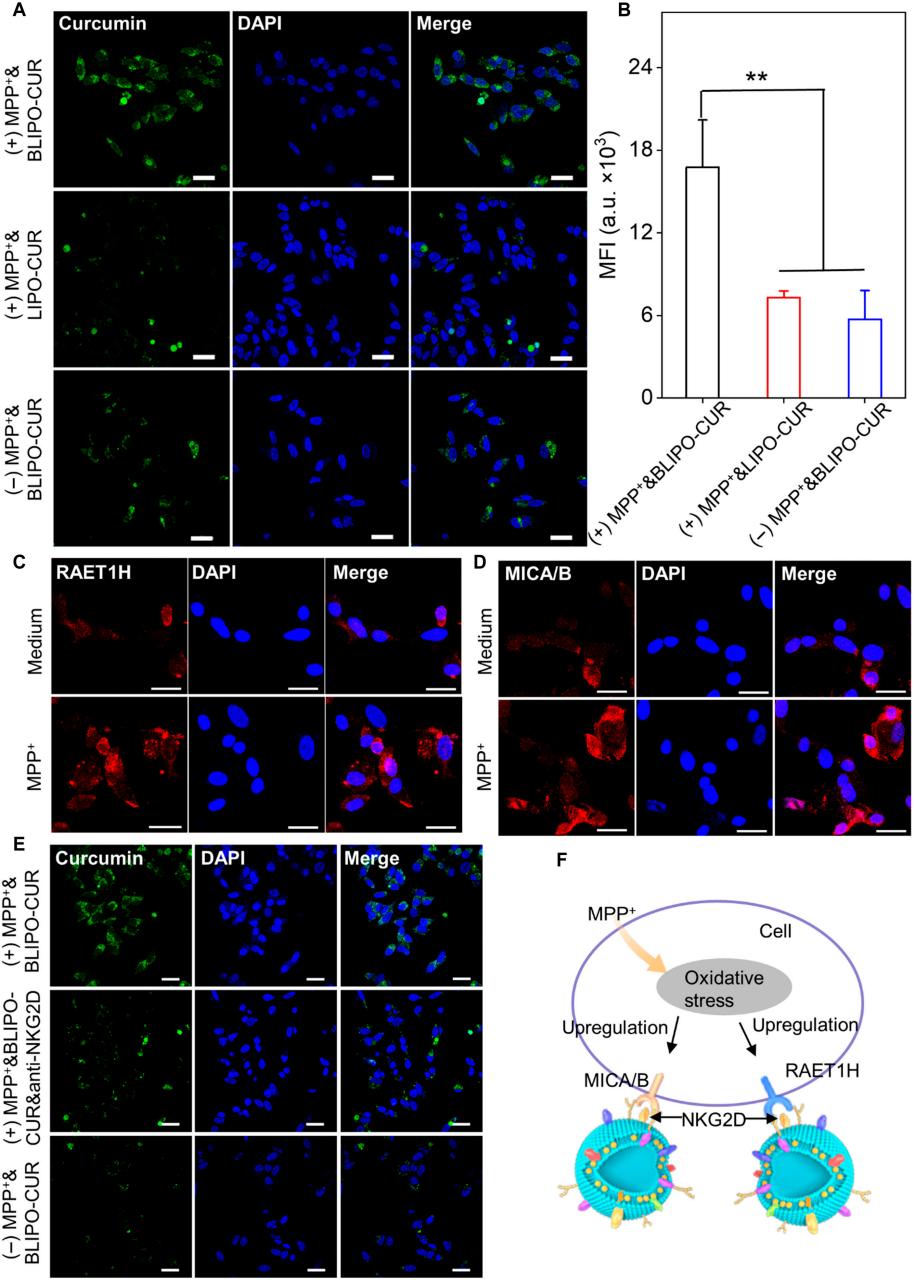
Evaluation of the ability of BLIPO-CUR to actively target broken neurons in vitro. (A) Confocal fluorescence images of SH-SY5Y cells with cotreatment of MPP^+^ and BLIPO-CUR, cotreatment of MPP^+^ and LIPO-CUR, and treatment of BLIPO-CUR, respectively. Scale bars, 25 μm. (B) Quantification of mean fluorescence of curcumin in (A). MFI, mean fluorescence intensity. (C) RAET1H and (D) MICA/B ligands expressed on SH-SY5Y cells with or without MPP^+^ treatment. Blue, staining of nuclei by DAPI; green, RAET1H or MICA/B labeling. Scale bars, 25 μm. (E) Confocal fluorescence images of SH-SY5Y cells incubated with BLIPO-CUR, MPP^+^-treated SH-SY5Y cells incubated with BLIPO-CUR, and MPP^+^-treated SH-SY5Ycells incubated with BLIPO-CUR blocked by anti-NKG2D antibody. Scale bars, 25 μm. (F) Illustration of the upregulated expression of RAET1H and MICA/B after MPP^+^ incubation and the procession showing BLIPO-CUR target broken neurons. *C*_BLIPO-CUR_ = *C*_LIPO-CUR_ = 8 μM. C_MPP_^+^ = 1 mM. All data are shown as the means ± SD; **P < 0.01; one-way analysis of variance (ANOVA). a.u., arbitrary units.

Next, to assess the active targeting potential of BLIPO-CUR, we established a cellular model of PD using SH-SY5Y cells treated with methyl-4-phenylpyridinium (MPP^+^) [25]. After incubation with MPP^+^-induced SH-SY5Y cells, the BLIPO-CUR-treated group exhibited a brighter green fluorescence signal than the LIPO-CUR-treated group, indicating that much more BLIPO-CUR was taken up by the mimic damaged neuronal cells (Fig. 3A). In addition, normal SH-SY5Y cells incubated with BLIPO-CUR also exhibited lower green fluorescence signals than that of MPP^+^-treated SH-SY5Y cells. The quantitative analysis showed that BLIPO-CUR-treated mimic damaged neuronal cells possessed the highest fluorescence signals than other groups, which was about 2.9 and 1.8 times higher than the LIPO-CUR-treated group and BLIPO-CUR-treated normal SH-SY5Y cells, respectively (Fig. 3B). These results demonstrated the specific targeting ability of BLIPO-CUR to mimic damaged neuronal cells.

One of the primary mechanisms related to PD is oxidative stress that is related to enhanced ROS generation and impaired antioxidant status [26]. Note that the MHC class I polypeptide-related sequence A and B (MICA/B) and the member of retinoic acid early transcripts-1 (RAET1H) both of which were ligands of Natural killer group 2 member D (NKG2D), have been reported to be upregulated on the surface when cells were under oxidative stress status and they can strongly interact with NKG2D expressed by NK cells [27–29]. To further investigate the active targeting mechanism of the BLIPO-CUR, we analyzed the expression of RAET1H and MICA/B proteins on SH-SY5Y cells before and after treatment by MPP^+^ that could induce oxidative stress. As demonstrated in Fig. 3C and D and Fig. S5A and B, the expression level of RAET1H and MICA/B proteins was dramatically upregulated in the MPP^+^-treated cells as compared with normal cells, consistent with the results from flow cytometry analysis (Fig. S6A to D). Next, we confirmed the retained NKG2D on BLIPO-CUR by Western bot (Fig. S7). Therefore, we reasoned that the active targeting ability of BLIPO-CUR originated from the NKG2D on the CMFs. To verify this, we conducted the confocal imaging of SH-SY5Y cells with different treatments [(+)MPP^+^&BLIPO-CUR, (+)MPP^+^&BLIPO-CUR&anti-NKG2D, and (−)MPP^+^&BLIPO-CUR]. It was observed that the fluorescence intensity of SH-SY5Y cells incubated with BLIPO-CUR was low. In addition, the fluorescence intensity in the MPP^+^-treated SH-SY5Y cells incubated with BLIPO-CUR after being blocked by anti-NKG2D antibody dramatically decreased as compared with the unblocked group. These results further confirmed that the NKG2D on the surface of the BLIPO-CUR is devoted to its active targeting ability for broken SH-SY5Y cells (Fig. 3E and Fig. S5C).

In addition, the anti-immune phagocytic ability of BLIPO-CUR was evaluated in Raw 264.7 cells. The result showed that little BLIPO-CUR was internalized by Raw 264.7 cells (Fig. S8A). In addition, the fluorescence intensity of BLIPO-CUR in Raw 264.7 cells was 2.9-fold weaker than that of LIPO-CUR-treated cells from flow cytometry (Fig. S8B and C), suggesting the potent ability of BLIPO-CUR to escape from endocytosing by macrophages.

### In vitro biocompatibility and neuroprotective effect of BLIPO-CUR

**Fig. 4.**
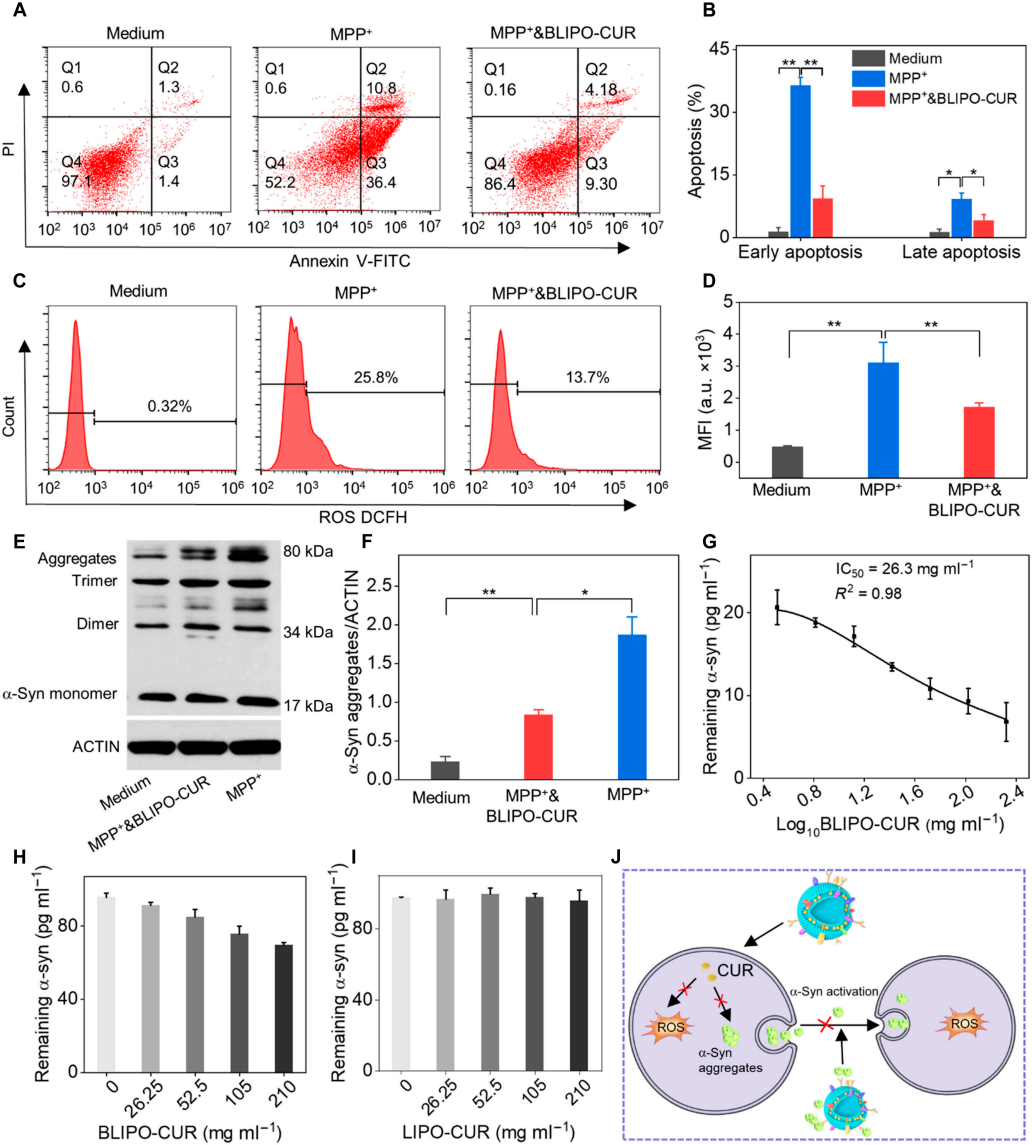
Protective effect and mechanism of BLIPO-CUR against the neurotoxicity induced by MPP^+^ in SH-SY5Y cells. (A) Analysis of cell apoptosis by flow cytometry. Early apoptotic cells are present in Q3 (Annexin V-FITC-positive and PI-negative) and late apoptotic cells are present in Q2 (both annexin V-FITC and PI-positive) (B) Calculated apoptosis (%) of early and late apoptotic cells. (C) Flow cytometry analysis of ROS-positive SH-SY5Y cells under different treatments. (D) Quantitative analysis of the intensity of DCFH fluorescence in each group of (C). (E) Representative Western blot results show the expression of α-syn species in SH-SY5Y cells with different treatments. (F) Statistical analysis of α-syn aggregates in (E). (G) Binding profile of BLIPO-CUR with α-syn (20 pg ml^−1^). Nonlinear regression fitting with inhibitory dose model (variable–slope model) was used to process the data. (H) BLIPO-CUR and (I) LIPO-CUR dose-dependent inhibition of α-syn released by SH-SY5Y cells. (J) Proposed mechanism of BLIPO-CUR against the α-syn aggregates, clearing the ROS and inhibiting the seeding of α-syn among cells. All results are presented as the means ± SD; *P < 0.05; **P < 0.01; one-way ANOVA; *n* = 3.

The biocompatibility and neuroprotective effect of BLIPO-CUR were investigated for further application. The cell viabilities were over 95% even at 8 μM curcumin (Fig. S9A and C), indicating good biocompatibility of BLIPO-CUR and LIPO-CUR. Next, we evaluated the neuroprotective effect of the BLIPO-CUR using MPP^+^-induced SH-SY5Y cells. As demonstrated in Fig. 4A and B, the percentage of early and late apoptotic cells increased to approximately 36.4% and 10.8% after MPP^+^ treatment, respectively. Interestingly, the apoptosis rate of cells pretreated with BLIPO-CUR was around 9.3% and 4.18%, which were much lower than those of only the MPP^+^-treated group. This result indicated that BLIPO-CUR could protect SH-SY5Y cells from the neurotoxicity induced by MPP^+^. The higher cell viability of SH-SY5Y cells after coincubation with BLIPO-CUR and MPP^+^ further confirmed this protection ability (Fig. S9B). In addition, LIPO-CUR also demonstrated a slighter protective effect compared with BLIPO-CUR (Fig. S9D).

Then, we evaluated the interaction between BLIPO-CUR and ROS, since ROS plays an important role in the apoptosis of neurons. As demonstrated in Fig. 4C and D, ROS-positive cells increased to 25.8% in the MPP^+^-treated group and decreased to 13.7% in SH-SY5Y cells cotreated by BLIPO-CUR and MPP^+^. It was validated that the BLIPO-CUR could reduce the ROS level in SH-SY5Y cells induced by MPP^+^. Besides ROS, excess α-syn species generated by the neurons could not only form α-syn aggregates in the neurons and induce apoptosis but could also be released from the neurons to damage the surrounding neurons and astrocytes [30–32]. Therefore, we further investigated the effect of BLIPO-CUR on α-syn species. It was found that the BLIPO-CUR could dramatically reduce the α-syn aggregates in the MPP^+^-induced SH-SY5Y cells (Fig. 4E and F). Note that TLR4 that is expressed on the surface NK cells could absorb α-syn, which plays a role in the pathology of PD [33]. Interestingly, TLR4 expressed on NK cell membranes was also retained in BLIPO-CUR rather than LIPO-CUR (Fig. S10). Thus, the ability of BLIPO-CUR to clear the α-syn was further measured, showing half-maximal inhibitory concentration (IC_50_) value of 26.3 mg ml^−1^ (Fig. 4G). In addition, the exocytosis α-syn from the SH-SY5Y cells induced by MPP^+^ could be cleared by the BLIPO-CUR, whereas LIPO-CUR could not clear the α-syn (Fig. 4H and I). Overall, the BLIPO-CUR performed the neuroprotective effect through scavenging ROS and decreasing α-syn species (Fig. 4J).

### BLIPO-CUR delivered via MLV route

**Fig. 5.**
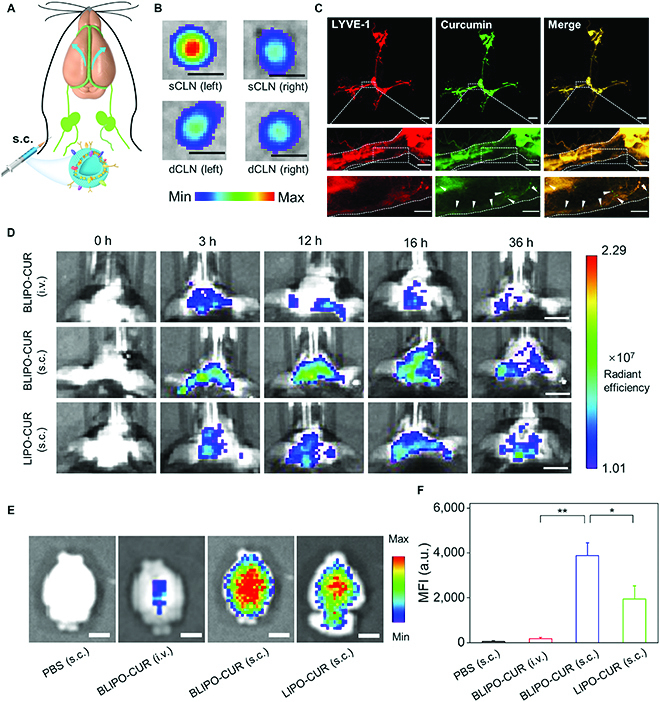
In vivo fluorescence imaging of BLIPO-CUR drained through MLVs in healthy mice. (A) Illustration of MLV route-mediated delivery of BLIPO-CUR into brain tissues. (B) The representative ex vivo fluorescence images of CLNs of the BLIPO-CUR (subcutaneously) group. sCLN, superficial cervical lymph node. Scale bars, 1 mm. (C) Representative images of MLVs labeled by Lyve-1 (red) and curcumin (green) in mice treated with subcutaneous injection of BLIPO-CUR. Scale bars, 1 mm (top), 200 μm (middle), and 100 μm (bottom). (D) Real-time fluorescence images of the mice before and after intravenous (i.v.) injection of BLIPO-CUR, subcutaneous injection of BLIPO-CUR, and subcutaneous injection of LIPO-CUR. Scale bars, 5 mm. (E) Representative in vivo imaging system (IVIS) images of the brains of the mice ex vivo at 12 h after injections. Scale bars, 5 mm. (F) Semiquantitative results of the brain of mice ex vivo in (E). All data are shown as means ± SD; *P < 0.05; **P < 0.01; one-way ANOVA.

Different from the blood delivery system, MLVs, another set of the circulatory system of the brain offers an opportunity to drain the drugs into the brain [13]. Next, we investigated the feasibility of delivering BLIPO-CUR into the brain of mouse via the MLV route. The BLIPO-CUR was administrated into the mouse through subcutaneous administration near the local lymph nodes at the neck (Fig. 5A). To identify the brain lymphatic vasculature-mediated delivery, especially the original drainage of BLIPO-CUR from the injection site to nearby lymph nodes, we isolated and imaged the cervical lymph nodes including deep cervical lymph nodes (dCLNs) connecting to MLVs at 4 h after injection (Fig. S11). As illustrated in Fig. 5B, the CLNs including dCLNs were lighted up, demonstrating BLIPO-CUR infiltrated into the draining lymph nodes of MLVs. In addition, the MLVs of mouse were collected to further investigate whether the BLIPO-CUR was drained in MLVs after initial drainage into the local lymph nodes (Fig. 5C). The MLVs were labeled by the lymphatic vessel endothelial receptor-1 (Lyve-1) (red) to distinguish them from blood vessels. The green fluorescence signal of BLIPO-CUR was observed, which merged well with the red fluorescence signal (Lyve-1). There were also some independent particles with green fluorescence (indicated by the white arrowheads) in MLVs that can be detected, which may be the aggregates of BLIPO-CUR. These results demonstrated that BLIPO-CUR could be transported via the MLV route. To explore the efficiency of BLIPO-CUR entering the brain through MLV route-mediated delivery, BLIPO-CUR was intravenously and subcutaneously administrated into the mouse at the neck site, respectively. The fluorescence signals of the brain were monitored using in vivo imaging system over time (Fig. 5D). The fluorescence of subcutaneous injection of BLIPO-CUR-treated mouse remained at a higher level at least 3 to 36 h compared to that of intravenous injection of BLIPO-CUR-treated mouse, indicating that the MLVs could be a more efficient delivery route to the brain than the conventional intravenous injection.

In addition, it observed that the fluorescence signals of subcutaneous injection of BLIPO-CUR-treated mice were much higher than that of subcutaneous injection of LIPO-CUR-treated mice, indicating that the biomimetic strategy enhanced the entering efficiency of liposomes into the brain through MLV route. To confirm this, we collected the brain tissue in different treated groups at 12 h after injection for ex vivo imaging. The brains of PBS-treated mice nearly did not show fluorescence signal; it implied that it could exclude the interference from the brain itself. It can be observed that the brain of the mouse treated by subcutaneous injection of BLIPO-CUR showed the strongest fluorescent signal, which was about 25- and 2.3-fold higher than that of the brain of the mouse treated by subcutaneous injection of BLIPO-CUR and LIPO-CUR, respectively (Fig. 5E and F). In addition, the loss-of-function experiments using MLV-defective mice were carried out. Under the guidance of previous reports [34], a photosensitizer named Visudyne was injected into the cisterna magna of the mice and further irradiated by laser to ablate the MLVs. It was observed that the fluorescence signal of the “Visudyne+laser” group was much lower than the “Visudyne” group and “laser” group (Fig. S12), further implying the function of MLVs to deliver BLIPO-CUR. All these results verified the high delivery efficiency of biomimetic liposomes to the brain through MLV routes.

### In vivo PD therapy

**Fig. 6.**
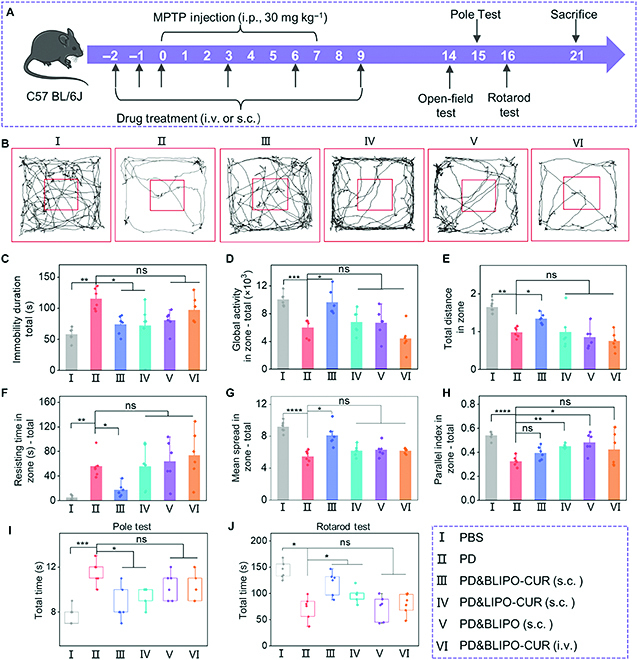
Protective effects of BLIPO-CUR delivered through MLVs on MPTP-induced mouse models of PD. (A) Schematic illustration of the schedule for PD mouse model establishment and drug treatments. i.p., intraperitoneal. (B) Open-field test. Representative track test sheets. (C to H) Parameter analysis shows the change in global activity, resting time, total distance, mean speed, duration of immobility, and parallel index in the open-field test. (I) Pole test. The average time of mice to climb the whole pole equipment. (J) Rotarod test. The averaged holding times of mice on the rotarod. All data are shown as means ± SD; *n* = 6 animals for each group. *P < 0.05; **P < 0.01; ***P < 0.001; ****P < 0.0001; one-way ANOVA. ns, not significant.

We subsequently evaluated in vivo therapeutic efficacy of BLIPO-CUR in mouse PD models, which was induced by 1-methyl-4-phenyl-1, 2, 3, 6-tetrahydropyridine (MPTP) (Fig. 6A) [35]. The animals were randomly divided into 6 different treatments: (I) normal mice treated by PBS; (II) PD mice without treatment; (III) PD mice treated by subcutaneous injection of BLIPO-CUR into MLVs; (IV) PD mice treated by subcutaneous injection of LIPO-CUR into MLVs; (V) PD mice treated by subcutaneous injection of BLIPO vesicles into MLVs; and (VI) PD mice treated by intravenous injection of BLIPO-CUR. Then, a series of behavioral tests mainly including an open-field test, pole test, and rotarod test were further conducted to assess the therapeutic effect.

In the open-field test, the PD mice treated with subcutaneous injection of BLIPO-CUR showed similar motion behavior in the total zone and residence time in the central grid to normal mice, which was much better than the PD mice and other groups with different treatments (Fig. 6B and Fig. S13). The quantitative data including global activity, total distance, mean speed, immobility duration, resting time, and the parallel index of the PD mice with different treatments were improved as compared with PD mice without treatments. It indicated that the subcutaneous injection of BLIPO-CUR showed the best efficiency in ameliorating the anxiety status and memory dysfunction of the PD mice than other methods, suggesting the good therapeutic effect of BLIPO-CUR delivered via MLV drainage route (Fig. 6C to H). In addition, the PD mice treated with subcutaneous injection of BLIPO-CUR had reduced the time of climbing pole, confirming the PD therapeutic effect of BLIPO-CUR (Fig. 6I). In the rotarod test, the PD mice treated with subcutaneous injection of BLIPO-CUR exhibited an average holding time of about 130 s, which was 60 s longer than the PD mice without treatment. However, the average holding time for PD mice treated by subcutaneous injection of LIPO-CUR and BLIPO and intravenous injection of BLIPO-CUR was about 100, 85, and 95 s, respectively, which were all shorter than that of subcutaneous injection of the BLIPO-CUR-treated group (Fig. 6J). It showed that all treatments affected the behaviors of PD mice and the treatment of subcutaneous injection of BLIPO-CUR were demonstrated to be the best treatment option for PD mice. According to these behavioral tests, we concluded that the BLIPO-CUR delivered by MLV route was an effective method for in vivo PD therapy.

**Fig. 7.**
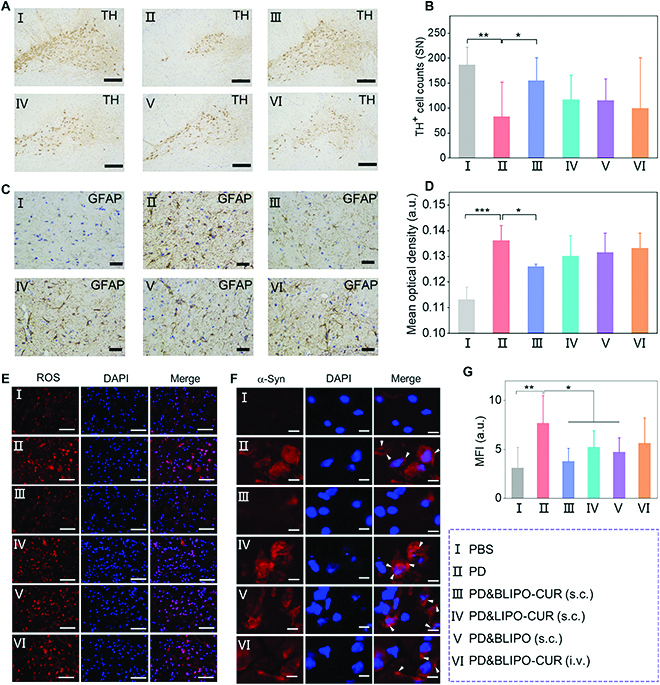
Protective effects of BLIPO-CUR drained through MLVs on the MPTP mouse model of PD. (A) Representative photomicrographs of TH^+^ dopaminergic neurons immunostaining in substantia nigra of the same animals. Scare bars, 25 μm. (B) Quantification of the surviving TH^+^ dopaminergic neurons in substantia nigra region. (C) Immunoreactions of the marker of activated astrocytes (GFAP), in substantia nigra of the same animals. Scare bars, 25 μm (D) Quantification of GFAP^+^ astrocytes in substantia nigra. (E) Representative fluorescence images of ROS (red) in substantia nigra. Cell nuclear: DAPI, blue. Scar bars, 100 μm. (G) Statistical analysis of the fluorescence intensity of ROS. (F) Typical fluorescence images of α-syn (red) in substantia nigra. Cell nuclear: DAPI, blue. Scale bars, 10 μm. All data are presented as means ± SD; *n* = 6 per group; *P < 0.05; **P < 0.01; ***P < 0.001; one-way ANOVA.

The PD mice were euthanized for the collection of tissue sections after different treatments. Histological analysis revealed that living dopaminergic neurons [labeled as tyrosine hydroxylase-positive (TH^+^)] in substantia nigra of the PD mice were remarkably reduced, which was markedly increased after treatment with subcutaneous injection of BLIPO-CUR (Fig. 7A and B). However, no obvious protective effect was observed in subcutaneous injection of LIPO-CUR- and BLIPO-treated groups, indicating that BLIPO-CUR possessed α-synergistic effect in PD therapy. More importantly, the intravenous injection of BLIPO-CUR-treated group showed no protective effect, indicating that the injection method was critical for PD therapy. As the astrocytes could be activated by α-syn species, the activated astrocytes labeled as glial fibrillary acidic protein-positive (GFAP^+^) were further analyzed using immunohistochemistry. It indicated that the expression of GFAP apparently decreased in the substantia nigra of the PD mice after treatment with subcutaneous injection of BLIPO-CUR (Fig. 7C and D). In addition, the generation of ROS and the expression of α-syn species of PD mice with different treatments were evaluated. The ROS in PD mice could be efficiently eliminated by subcutaneous injection of BLIPO-CUR (Fig. 7E and G). The signal of α-syn normally appeared in aggregates in the PD model (indicated by white arrowheads) also dramatically decreased in the mice treated by subcutaneous injection of BLIPO-CUR (Fig. 7F). These results confirmed that BLIPO-CUR delivered by MLV route offered a promising application prospect in neuroprotection and therapy of PD.

The in vivo biocompatibility of the BLIPO-CUR was further evaluated. The body weight showed an increase after treatment with BLIPO-CUR, indicating no obvious toxicity of BLIPO-CUR (Fig. S14). The hematoxylin and eosin staining of main organs obtained from the mice that were treated differently confirmed the biocompatibility of BLIPO-CUR (Fig. S14A). Importantly, BLIPO-CUR exhibited excellent biocompatibility and biosafety in the brain either, which is critical for materials in the treatment of brain disorders (Fig. S15B).

## Discussion

MLVs, as the efficient routes to delivery of drugs bypassing the BBB, have been proved by Chen’s group [20,21] and applied for glioma treatment. However, the shortage of active-targeting ability and being phagocytized by the immune cells reduced the delivery efficiency into the brain. In our study, NK CMFs biomimetic liposomes were demonstrated to enhance the active targeting ability and inhibit the immune cell phagocytosis, which was delivered via MLV route for PD therapy. The delivery efficiency to the brain of biomimetic liposomes exhibited ~2 times higher than that of the liposome without NK CMF modification and ~20 times higher than that of being delivered through the brain–blood system. In addition, the biomimetic liposome could clear the α-syn species that aggregated in the neurons to induce apoptosis and exocytosis from the neurons to damage the surrounding neurons and astrocytes. Moreover, they could consume ROS, leading to neuroprotective effects on neurons. Thus, our study not only opens new opportunities to advance biomimetic nanomedicines in PD therapy but also provides a new administration route for various brain diseases.

## Materials and Methods

### Materials

Curcumin (purity ≥ 65%), MPP^+^ iodide, and ROS staining solution were obtained from Sigma-Aldrich (St. Louis, MO, USA). Soybean PC (l-α-lecithin) was procured from Ruixi Biotech Co. Ltd. (Xi’an, China). Cholesterol was procured from Avanti (Alabaster, AL, USA). Penicillin–streptomycin was obtained from Hyclone (USA). High-glucose Dulbecco’s modified Eagle’s medium (DMEM), trypsin EDTA, and fetal bovine serum were obtained from Gibco Life Technologies (AG, USA). The 4,6-diamidino-2-phenylindole (DAPI), annexin V–fluorescein isothiocyanate (FITC) apoptosis detection kit, Coomassie blue, radioimmunoprecipitation assay lysis buffer, bicinchoninic acid protein kit (BCA), and ROS assay kits were procured from Beyotime Biotechnology (China). MPTP-HCl and α-syn enzyme-linked immunosorbent assay kit were obtained from Lianshuo Chuangxiansheng Biotechnology Co. Ltd. (China). Monoclonal RAET1H antibody and monoclonal MICA/B antibody were purchased from Signalway Antibody (CA, USA). The NK cell isolation kit was obtained from Miltenyi Biotec (German). Rabbit anti-Lyve-1 antibody, mouse anti-TLR4 antibody, mouse anti-TH antibody, mouse anti-GFAP antibody, and rabbit anti-α-syn antibody were purchased from Servicebio (China).

### Isolation of NK cells membrane

The isolated NK cells from the spleens of mice were suspended in ice-cold Tris MgSO_4_ buffer solution and, afterward, were extruded 20 times through a mini-extruder to break the cells. The mixture that was in 1 M sucrose concentration situation was centrifuged at 2,000*g* for 10 min at 4 °C. After the centrifugation for another 30 min, the resulting CMFs were collected. The total protein contents of purified NK cell membrane were analyzed by BCA assay. The membrane materials were stored at −20 °C for further study.

### Preparation and characterization of BLIPO-CUR

The NK cells were isolated from the spleens of the female C57 BL/6J mice. CMFs were obtained from NK cells using hypotonic lysis, mechanical destruction, and centrifugation methods [36,37]. The protein concentration of the obtained membrane fragments was identified by BCA assay. Then, the thin-layer evaporation method was employed to prepare LIPO-CUR. Briefly, 100 mg of soybean phospholipid and 12.5 mg of cholesterol were dissolved in 10 ml of chloroform, and 5 mg of curcumin was dissolved in 5 ml of methyl alcohol, respectively. They were then added to the same flask, and the mixture was dried under a vacuum using a rotary evaporator to form a thin film at 27 °C. The thin and dried phospholipid blends were hydrated in PBS containing CMFs at a ratio of 1:600 (w/w) protein (the protein was quantified using BCA protein assay kit) to one phospholipid, followed by 5 cycles of freezing and thawing with liquid nitrogen and a water bath at 50 °C, respectively. The formed liposome dispersions were squeezed through polycarbonate membranes of 200-, 100-, and 50-nm pore size to produce small particles loaded with curcumin**.** Membrane materials were stored at −80 °C for future studies. Equations 1 and 2 were then used to calculate EE (%) and LE (%), respectively.

$$\mathrm{EE}\;(\%)=\frac{\text{Mass of CUR in NPs}}{\text{Mass of CUR in feed}}\times 100\%$$ (1)

$$\mathrm{LE}\;(\%)=\frac{\text{Mass of CUR in NPs}}{\text{Mass of CUR in NPs}+\text{Mass of NPs}}\times 100\%$$ (2)

The hydrodynamic size, zeta potential, and stability of the resulting NPs (LIPO-CUR and BLIPO-CUR) were determined by dynamic light scattering (Malvern). Transmission electron microscope was used to characterize the morphology of BLIPO-CUR. The fluorescence spectra and absorption spectra of free curcumin and BLIPO-CUR were observed using a fluorescence detector and an ultraviolet-visible detector, respectively. The concentration of curcumin was detected by spectrophotometry at an absorbance of 450 nm. Evaluation of drug leakage of free CUR and BLIPO-CUR in PBS containing 20% ethanol was recorded as well.

### Cellular uptake

The SH-SY5Y cells were cultured in an 8-well plate overnight. Then, they were treated with a fresh high-glucose DMEM with or without MPP^+^ (1 mM). After cultured for 24 h, the cells were cotreated with a fresh high-glucose DMEM containing LIPO-CUR and BLIPO-CUR, which was previously blocked by the anti-NKG2D antibody. The cells cultured in fresh medium containing BLIPO-CUR were taken as another control group. All the groups were incubated for a further 4 h. After being washed, fixed, and stained, the cells were subsequently imaged using a confocal fluorescence image system. Macrophage RAW 264.7 cells were continuously treated by LIPO-CUR and BLIPO-CUR for 4 h, and the cellular uptake was detected through confocal fluorescence image system and flow cytometry.

### In vitro cytotoxicity

To access the toxicity of BLIPO-CUR, SH-SY5Y cells were seeded into a 96-well plate overnight. These cells were then treated by BLIPO-CUR at various concentrations of curcumin ranging from 2 to 8 μM. After further incubation for 24 h, the cell viability was determined using CCK-8 assays.

### Binding analysis of α-syn by BLIPO-CUR

α-Syn (20 pg ml^−1^) was mixed with BLIPO-CUR under the concentrations (curcumin) from 0.4 to 2.4 mg ml^−1^. In a 37 °C incubator, the mixture was incubated for further 2 h and subsequently centrifuged at 10,000*g* for 15 min to separate the BLIPO-CUR. The concentration of rest α-syn in supernatant was measured by enzyme-linked immunosorbent assay kit.

### Therapeutic potential of BLIPO-CUR in MPP^+^-treated SH-SY5Y cells

SH-SY5Y cells were pretreated with BLIPO-CUR for 4 h and then cotreated with MPP^+^ (1 mM) for further 24 h. Then, the cell viability was identified by CCK-8 assays, and the percentage of dichloro-dihydro-fluorescein diacetate (DCFH-DA)-positive SH-SY5Y cells was detected by flow cytometry. Then, annexin V/propidium iodide (PI) staining and flow cytometry were applied to measure the apoptosis.

### Animals for in vivo studies

Female C57 BL/6J mice weighing about 20 g were from Beijing Vital River Laboratory Animal Technology Co. Ltd. (China) and kept at room temperature with free access to food and water. Animal experiments were performed following the criteria of Shenzhen Institution of Advanced Technology, Chinese Academy of Science Animal Care and Use Committee.

### In vivo fluorescence imaging

BLIPO-CUR was subcutaneously injected into the MLVs of normal mice. Mice with subcutaneous injection of LIPO-CUR (*C*_CUR_ = 1 mg kg^−1^) and intravenous injection of BLIPO-CUR were taken as controls. The fluorescence images were captured by an in vivo fluorescence imaging system (PerkinElmer).

### Processing of MLVs

At 12 h after subcutaneous administration of BLIPO-CUR, mice were perfused with 4% paraformaldehyde. Both right and left dCLNs and superficial cervical lymph nodes were extracted for imaging in vitro. Then, the skullcap was stripped and carefully polished. Finally, after the skull was fixed, dehydrated, and then rehydrated, the dura mater was gradually peeled off using ophthalmic tweezers.

### In vivo MPTP intoxication and drug treatment effect

The PD mouse model induced by MPTP was established according to previous reports. MPTP-HCl was injected intraperitoneally daily for 7 consecutive days at a dose of 30 mg kg^−1^. Subcutaneous administrations of BLIPO-CUR, LIPO-CUR, or BLIPO were performed 6 times in 12 days. Mice given an intravenous injection of BLIPO-CUR were taken as control. After the final injection of drugs, the mice were assessed using behavioral tests. The open-field test was carried out to assess the locomotor behavior of mice in the subsequent 3 min as described previously. Before testing, the mice were placed in the testing room for hours for adaptation. In pole test, the time taken by mice to climb from the top to the bottom of the pole equipment was recorded. In rotarod test, the mice were trained in the adaptive stage before MPTP treatments, and the speed of the equipment was set at 30 rpm. Then, the movement times of mice were measured after various treatments.

### ROS detection in substantia nigra

A spontaneous fluorescence-quenching reagent was added onto frozen slides and incubated for 5 min. Then, the ROS staining solution was added and incubated at 37 °C for 30 min. Then, the nucleus was counterstained using DAPI at 25 °C for 10 min. The slides were observed under a microscope, and images were collected by fluorescence microscopy.

### Immunofluorescence staining

The whole mounts and frozen sections were blocked by 3% bovine serum albumin for 40 min at 37 °C and then incubated with the primary antibody at 4 °C overnight. After decolorization by shaking in PBS (pH 7.4) thrice, the secondary antibodies were incubated at 25 °C for 2 h. Finally, the whole mounts and sections were mounted with the mounting medium containing DAPI.

### Histology and immunohistochemical staining

The heart, liver, spleen, lung, kidney, and brain of mice were cut into coronal slides for further procedure. Hematoxylin and eosin staining was used for the analysis of the morphology and inflammation, and immunohistochemistry was used to test the expression of the marker of activated astrocyte (GFAP) and the marker of live dopaminergic neuron (TH). Images of the above sections were captured using light microscopy (COIC, China).

## Data Availability

All data are available in the main text or the Supplementary Materials.

## Supplementary Materials

Supplementary 1 Fig. S1. Flow cytometry analysis of C57 BL/6J mouse splenocyte suspension. Fig. S2. Characterization the mixture of liposomes and CMFs. Fig. S3. Preparation and characterization of BLIPO-CUR. Fig. S4. Stability of the BLIPO-CUR in different media. Fig. S5. Quantitative analysis of flow cytometry results. Fig. S6. The expression of ligands for NKG2D receptors on MPP^+^-treated SH-SY5Y cells. Fig. S7. In vitro evaluation of immune escape ability of BLIPO-CUR. Fig. S8. In vitro cell toxicity and protective effect of BLIPO-CUR on SH-SY5Y cells. Fig. S9. Characterization of the TLR4 proteins. Fig. S10. The surgically isolated CLNs. Fig. S11. Open-field test. Fig. S12. Body weight of mice in all groups. Fig. S13. Biocompatibility evaluation of BLIPO-CUR. Table S1. Information of as-prepared BLIPO-CUR.
