# Supplementary material for: Delivery of Biomimetic Liposomes via Meningeal Lymphatic Vessels Route for Targeted Therapy of Parkinson’s Disease
Source: Research (Wash D C). 2023 Jan 30;6:0030. doi: 10.34133/research.0030 (PMC10076012; doi:10.34133/research.0030)
Supplement: Supplementary 1 — Fig. S1. Flow cytometry analysis of C57 BL/6J mouse splenocyte suspension. Fig. S2. Characterization the mixture of liposomes and CMFs. Fig. S3. Preparation and characterization of BLIPO-CUR. Fig. S4. Stability of the BLIPO-CUR in different media. Fig. S5. Quantitative analysis of flow cytometry results. Fig. S6. The expression of ligands for NKG2D receptors on MPP+-treated SH-SY5Y cells. Fig. S7. In vitro evaluation of immune escape ability of BLIPO-CUR. Fig. S8. In vitro cell toxicity and protective effect of BLIPO-CUR on SH-SY5Y cells. Fig. S9. Characterization of the TLR4 proteins. Fig. S10. The surgically isolated CLNs. Fig. S11. Open-field test. Fig. S12. Body weight of mice in all groups. Fig. S13. Biocompatibility evaluation of BLIPO-CUR. Table S1. Information of as-prepared BLIPO-CUR. [file research.0030.f1.docx]

**Delivery of Biomimetic Liposomes via Meningeal Lymphatic Vessels Route for Targeted Therapy of Parkinson's Disease**

Jing Liu^1,2^^†^, Duyang Gao^2†^, Dehong Hu^2^, Siyi Lan^2^, Yu Liu^2^, Hairong Zheng^2^*, Zhen Yuan^1^*, and Zonghai Sheng^2^*

***Corresponding author.** Hairong Zheng, hr.zheng@siat.ac.cn; Zhen Yuan, zhenyuan@um.edu.mo and Zonghai Sheng zh.sheng@siat.ac.cn

^1^Faculty of Health Sciences, University of Macau, Macau SAR 999078, P. R. China.

^2^Paul C. Lauterbur Research Center for Biomedical Imaging, Key Laboratory for Magnetic Resonance and Multimodality Imaging of Guangdong Province, Shenzhen Key Laboratory of Ultrasound Imaging and Therapy, CAS key laboratory of health informatics, Institute of Biomedical and Health Engineering, Shenzhen Institute of Advanced Technology, Chinese Academy of Sciences, Shenzhen, 518055, P. R. China.

**This PDF file includes:**

Figures. S1 to S13

Table S1

**
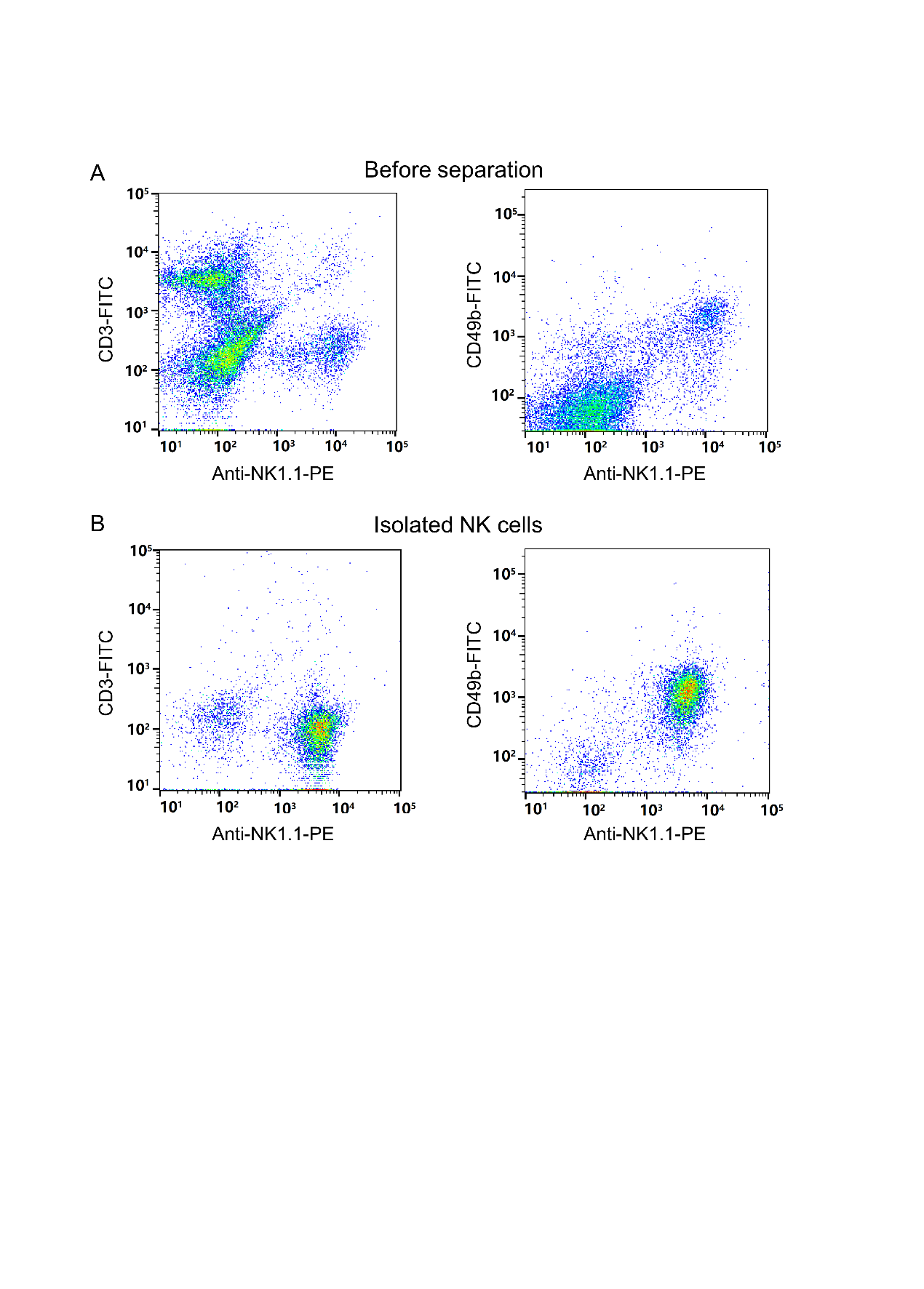
**

Figure S1. Flow cytometry analysis of C56 BL/6J mouse splenocytes suspension. (A) Before and (B) after isolating NK cells from spleen by flow cytometry.

**
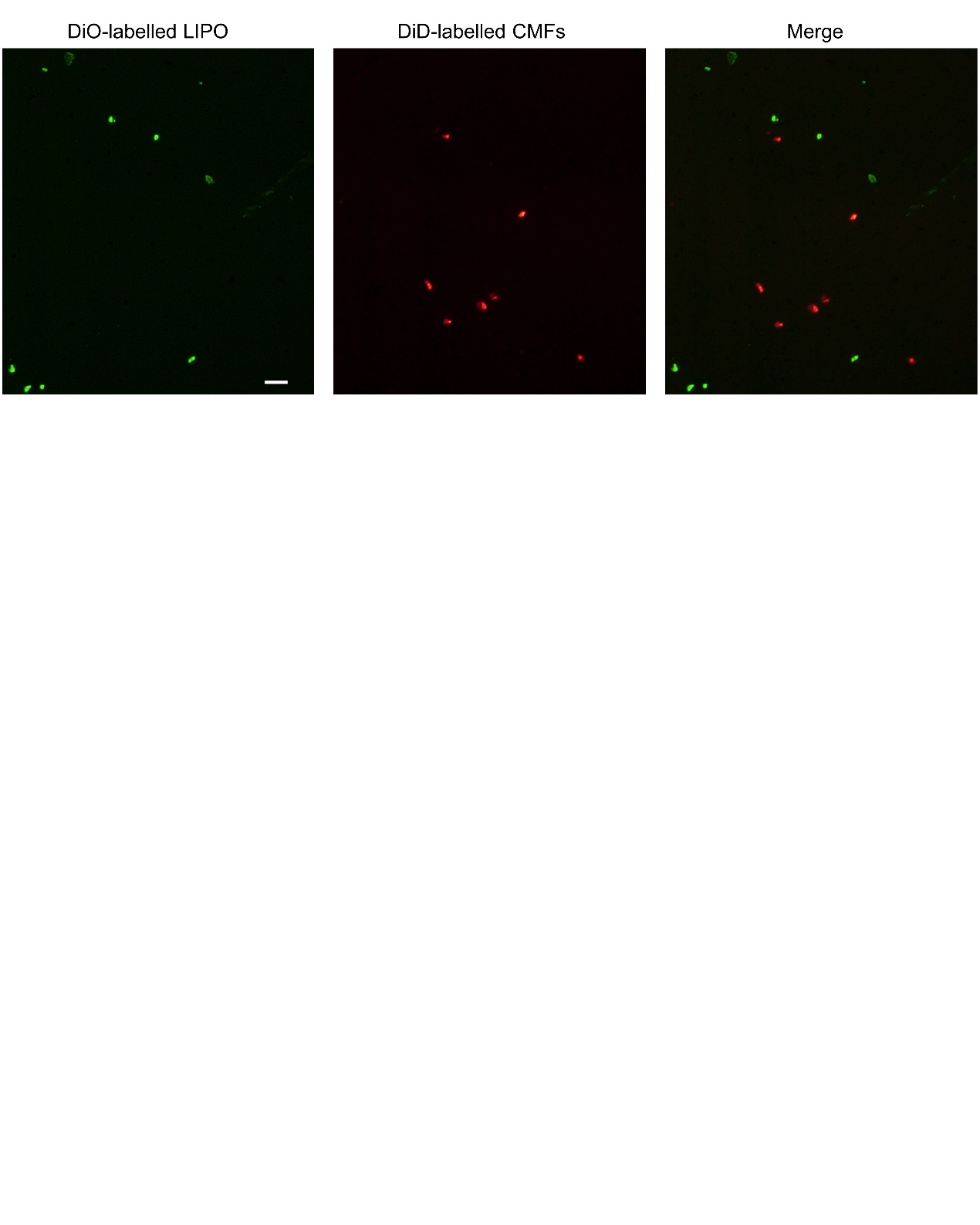
**

Figure S2. Characterization the mixture of liposomes and cell membrane fragments. Fluorescence images of the mixture of liposomes (DiO, Green) and cell membrane fragments (DiD, Red) subjected without freeze-thaw process. Scare bar, 2.5 μm.

**
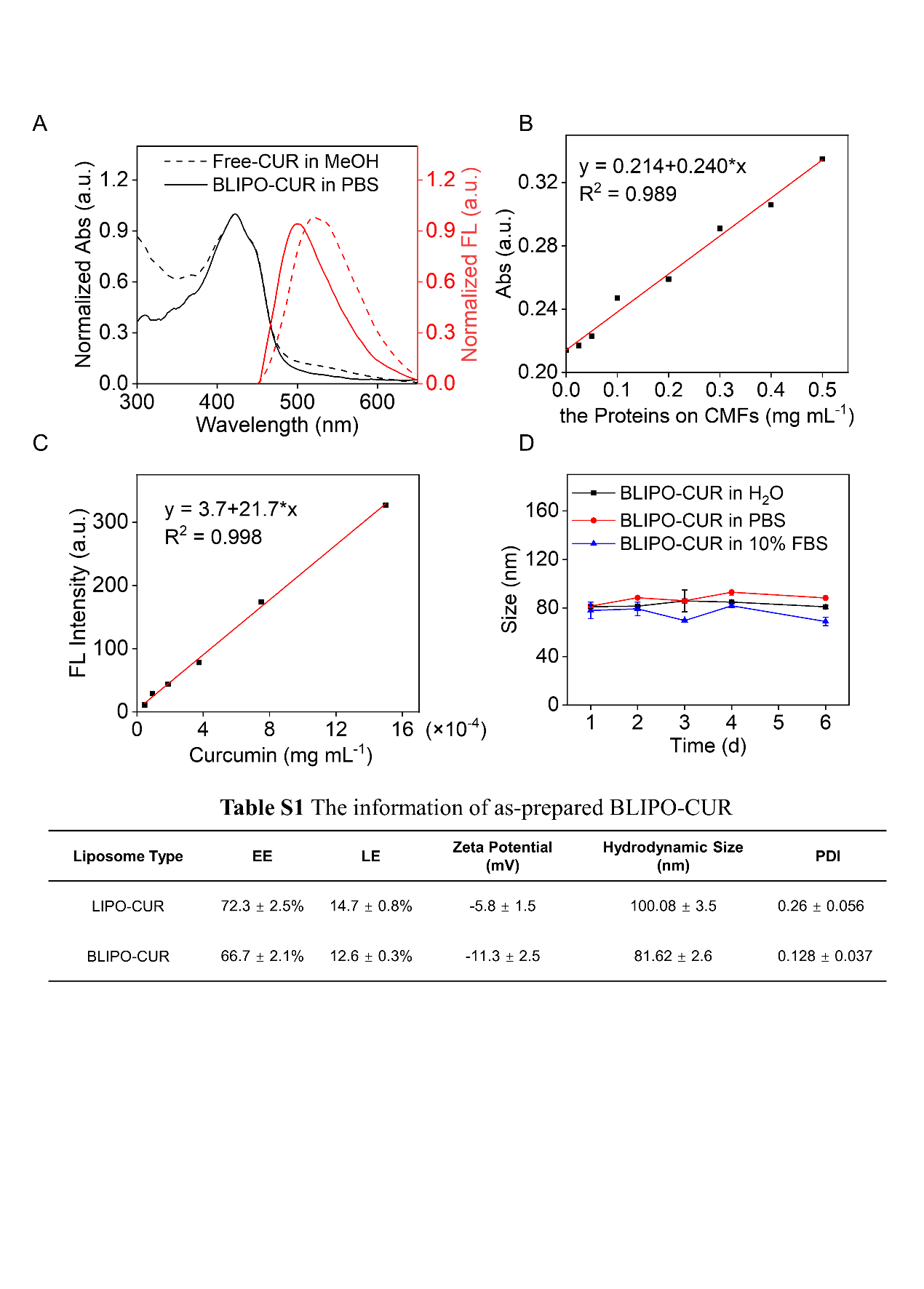
**

Figure S3. Preparation and characterization of BLIPO-CUR. (A) UV-vis absorption spectra (Black Curve) and fluorescence spectra (Red Curve) of free curcumin in MeOH and BLIPO-CUR in PBS. (B)The standard curve for measuring NK cell membrane proteins. (C) The standard curve for measuring curcumin in ethanol. (D) the hydrodynamic diameters of BLIPO-CUR in H_2_O, PBS and 10% FBS during a 6-day incubation.


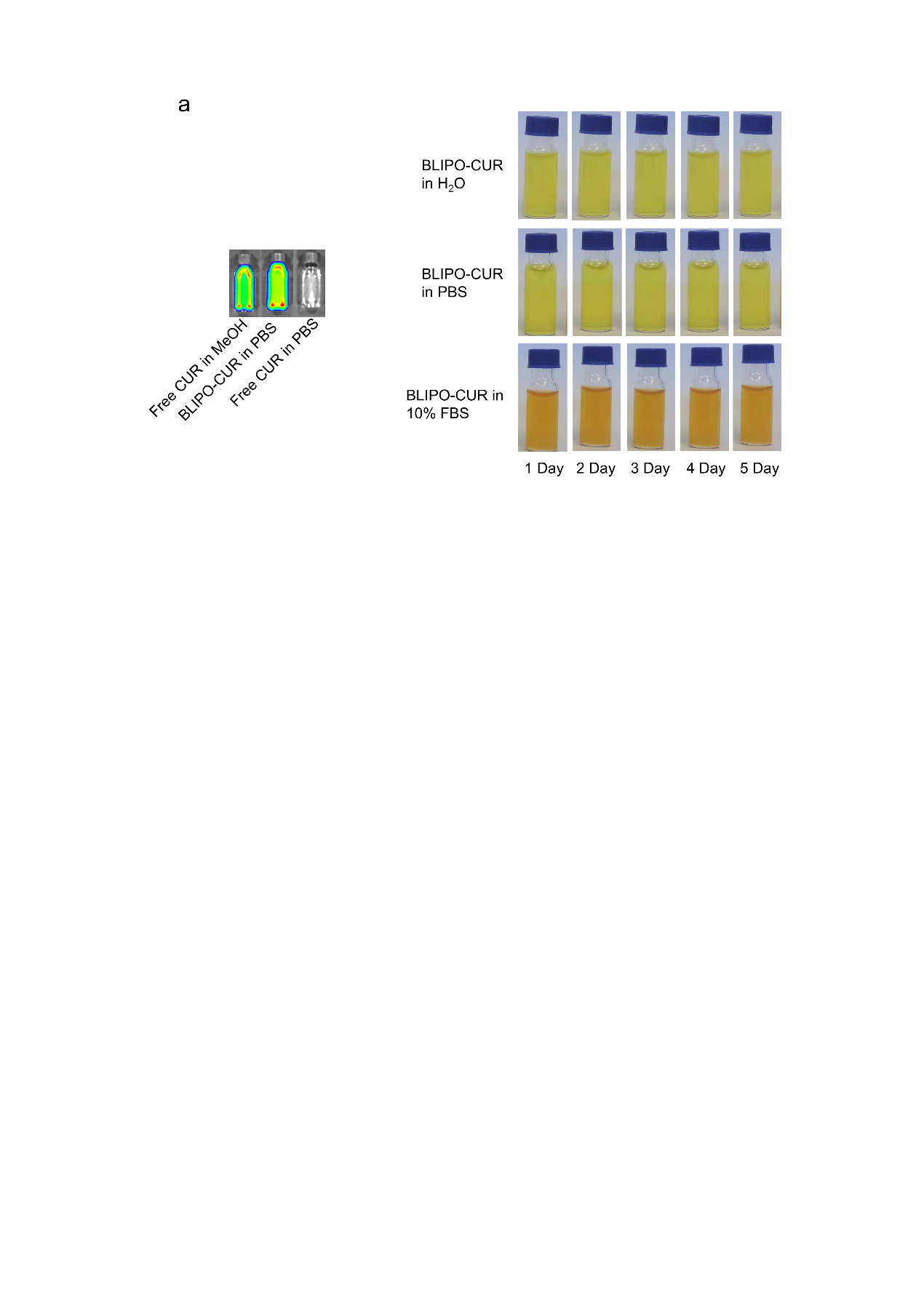
Figure S4. Stability of the BLIPO-CUR in different medium. The photo images of BLIPO-CUR in ddH_2_O, PBS (pH = 7.4) and 10% FBS during a 5-day storage in a 4 °C freezer.

**
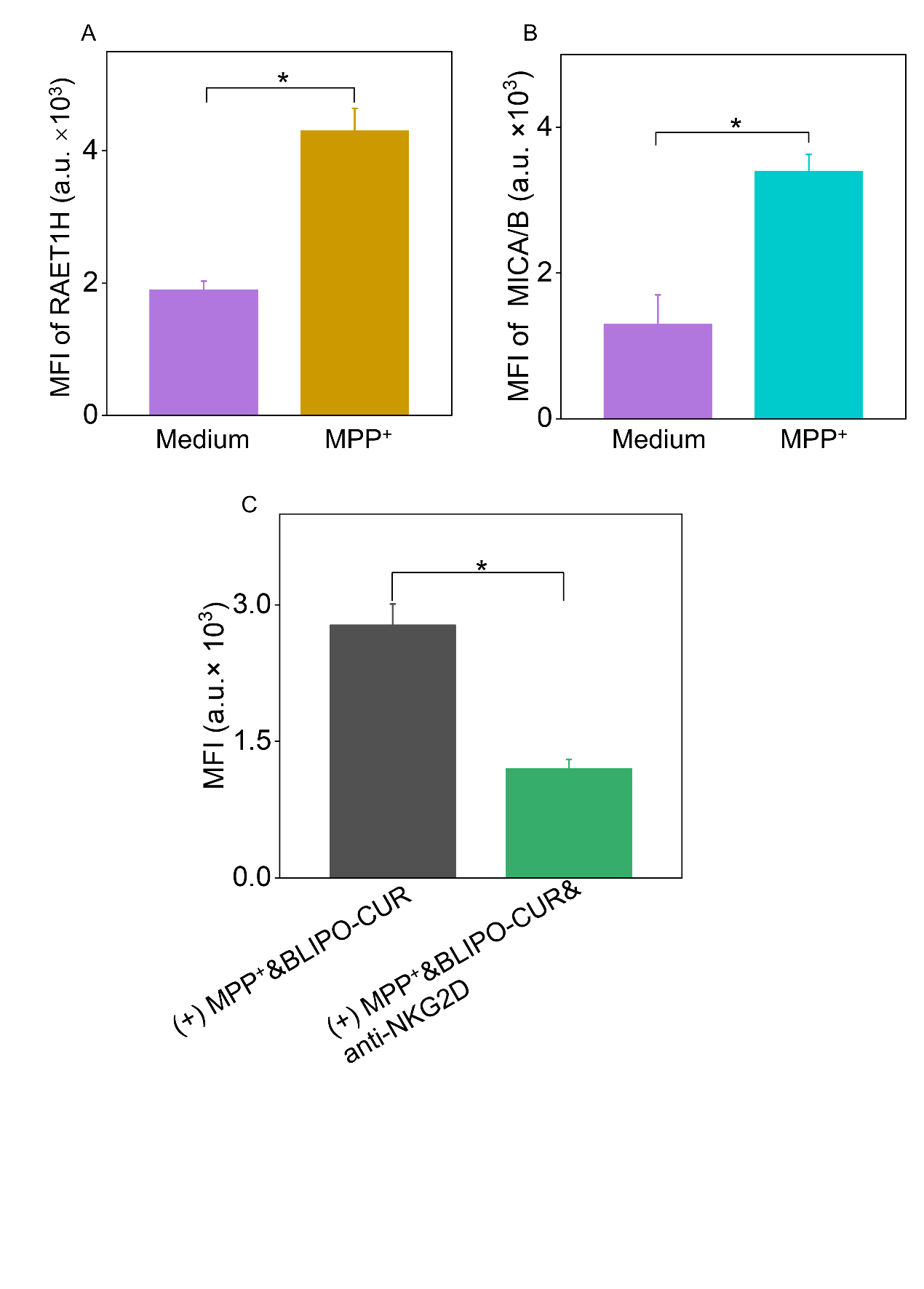
**

Figure S5. Quantitative analysis of flow cytometry results. Quantitative analysis of (A) RAET1H and (B) MICA/B expressed on SH-SY5Y cells treated with medium and MPP^+^. (C) Quantitative analysis of cellular uptake of BLIPO-CUR by MPP^+^-induced SH-SY5Y cells treated with BLIPO-CUR and BLIPO-CUR blocked by anti-NKG2D antibody. MFI. Mean fluorescence intensity. All the data represent mean ± S.D of three independent experiments. **p* < 0.05, One-way ANOVA.


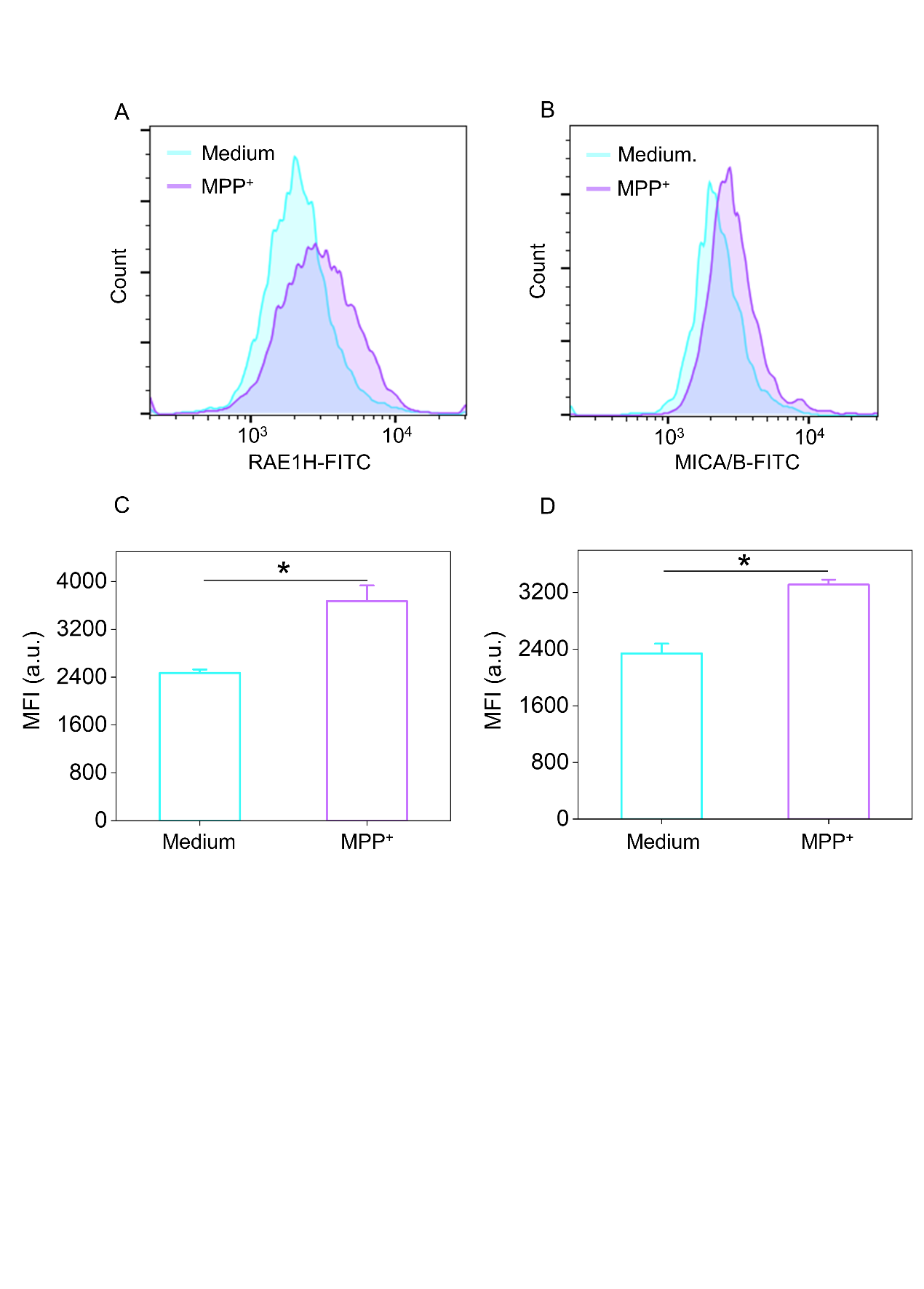


Figure S6. The expression of ligands for NKG2D receptors on MPP^+^-treated SH-SY5Y cells. (A-B) Flow cytometry analysis of RAE1H and MICA/B ligands on SH-SY5Y cells treated with MPP^+^ (1 mM) for 24 h. (C-D) RAE1H-FITC and MICA/B-FITC mean fluorescence intensity of cells with different treatments. All the data represent mean ± S.D of three independent experiments. **p* < 0.05, One-way ANOVA.

**
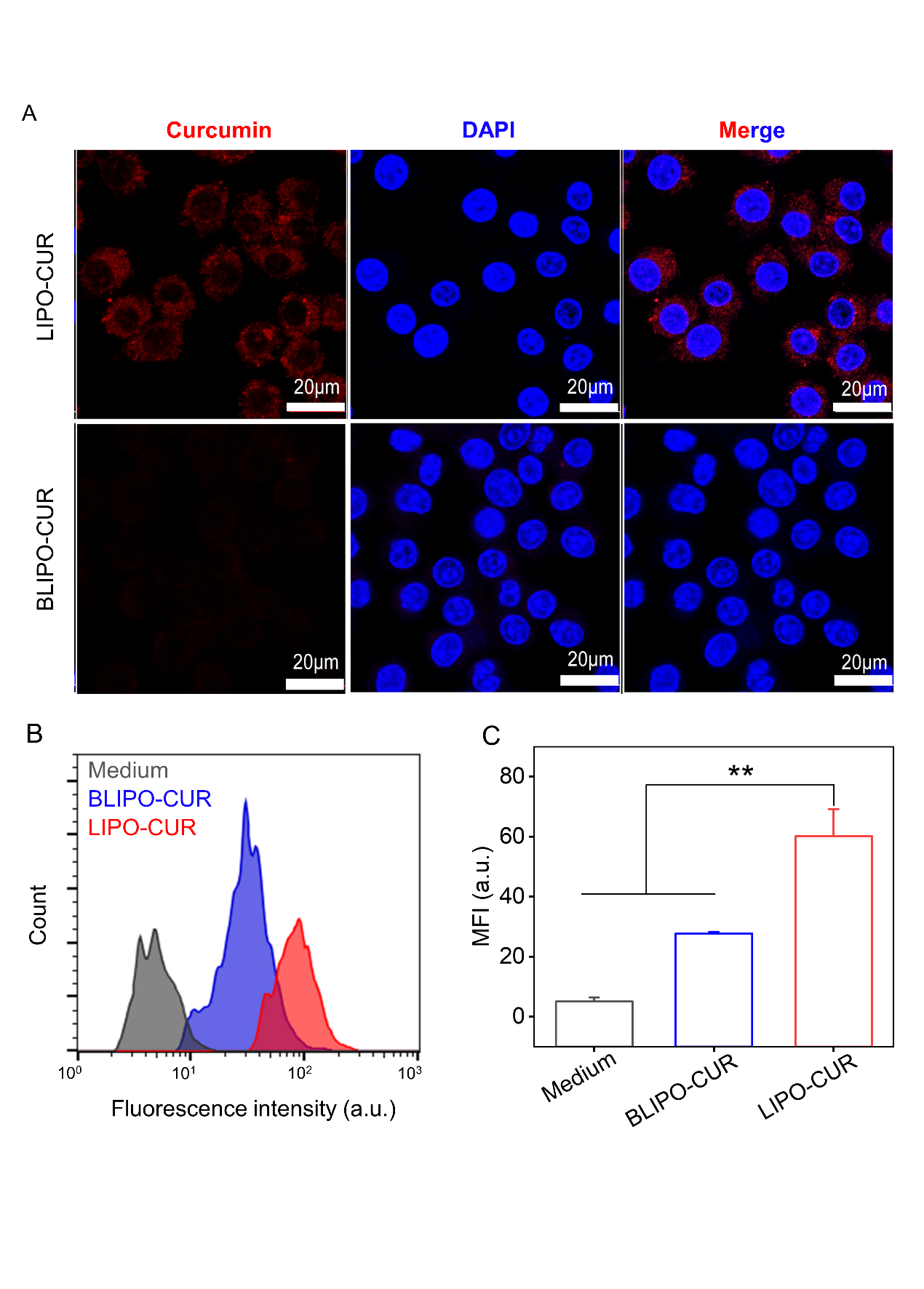
**

Figure S7. *In vitro* evaluation of immune escape ability of BLIPO-CUR. (A) CLSM images of Raw 264.7 cells treated with LIPO-CUR and BLIPO-CUR for 2 h. (B) Flow cytometry analysis of RAW264.7 cells treated with LIPO-CUR or BLIPO-CUR for 4 h. (C) Curcumin fluorescence intensity in Raw 264.7 cells. MFI, mean fluorescence intensity. All the data represent mean ± S.D of three independent experiments. ***p* < 0.01, One-way ANOVA.

**
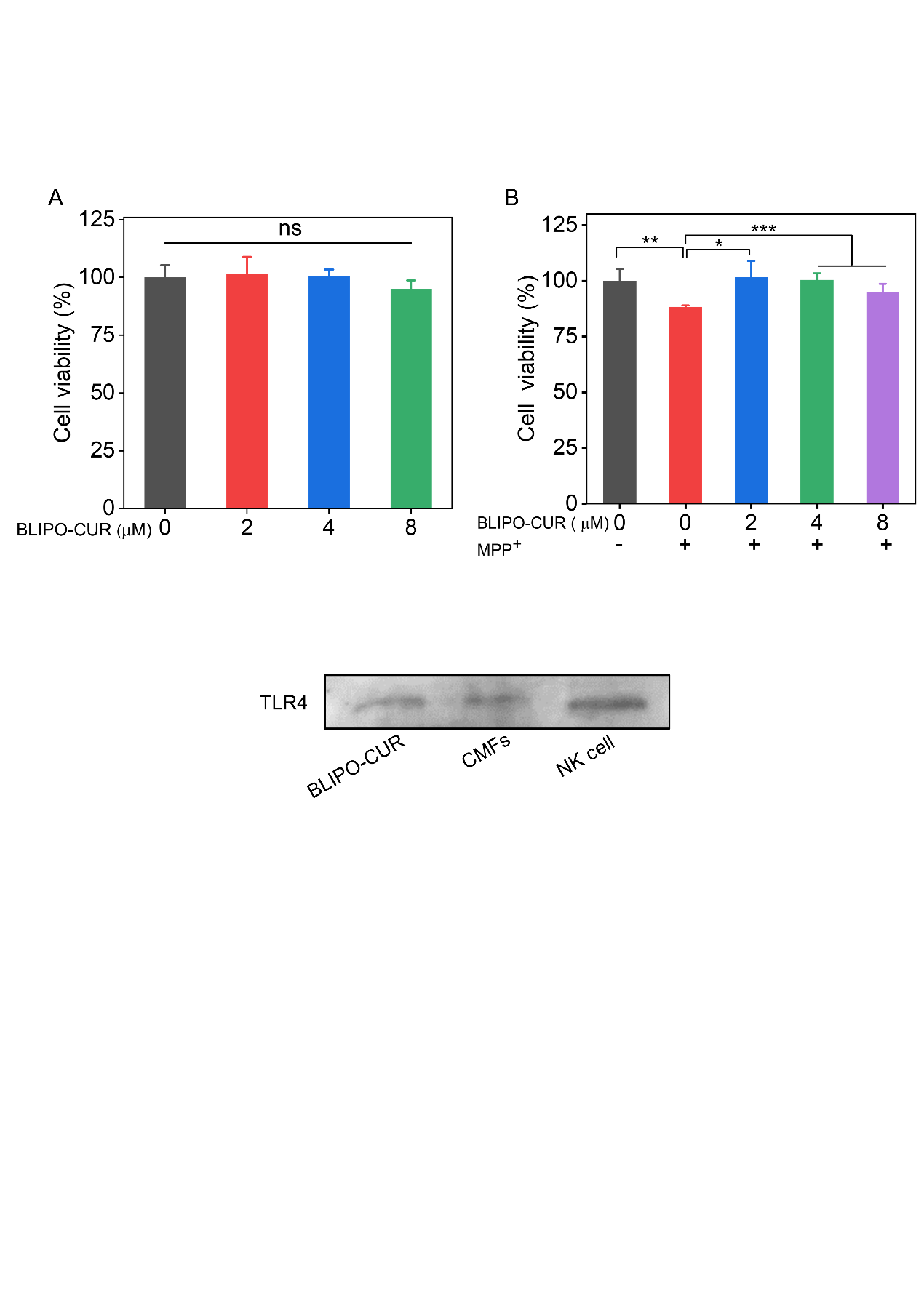
**

Figure S8. *In vitro* cell toxicity and protective effect of BLIPO-CUR on SH-SY5Y cells. (A) Cell viability of SH-SY5Y cells treated with BLIPO-CUR for 24 h and then tested by CCK8 assay. (B) Cell viability of SH-SY5Y cells that were treated with BLIPO-CUR for 4 h in advance and then co-treated with 1mM MPP^+^ for another 24 h. All the data represent mean ± S.D, n = 6. **p* < 0.05, ***p* < 0.01, ****p* < 0.001, One-way ANOVA.

**
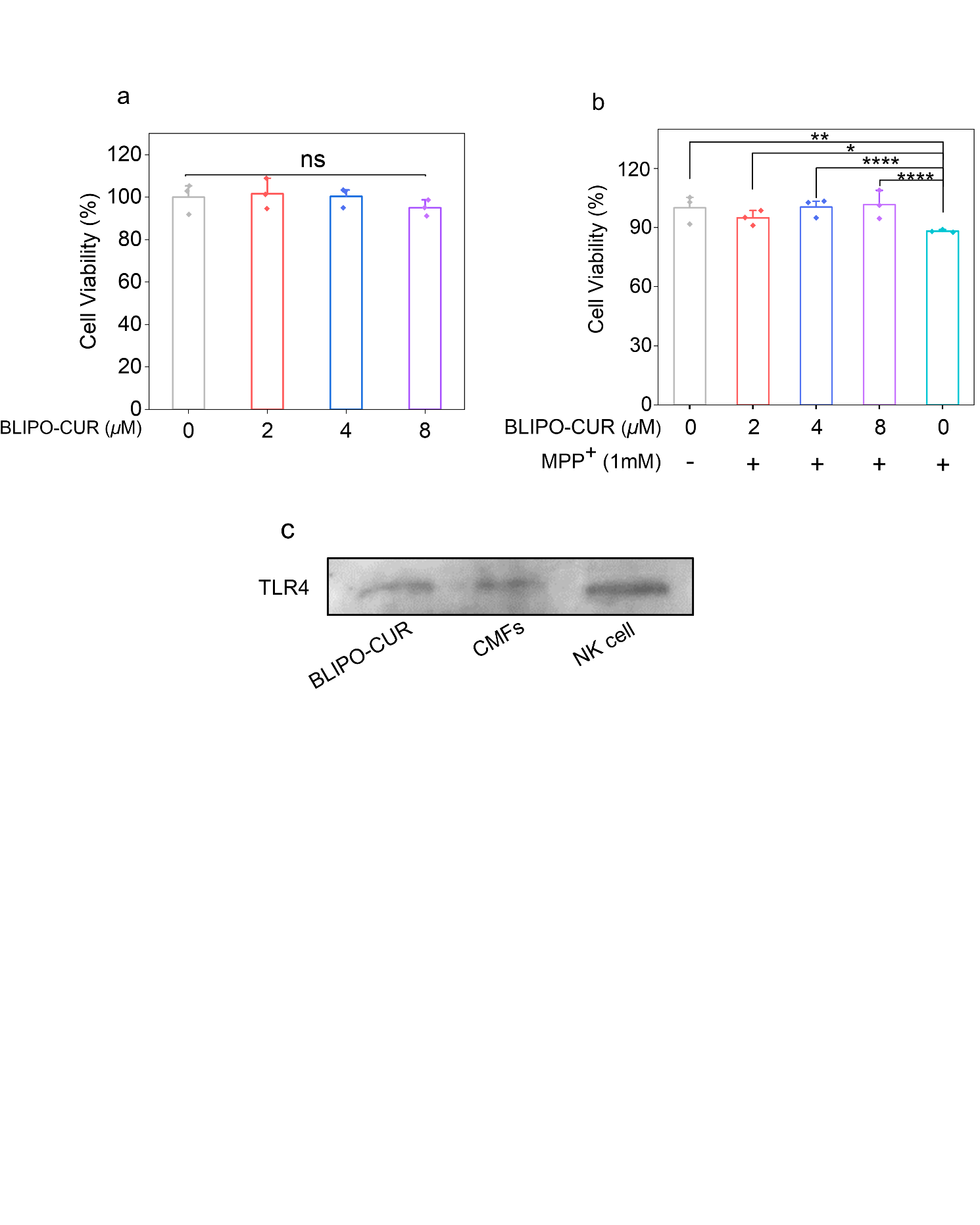
**

Figure S9. Characterization of the TLR4 proteins. Western blot result of TLR4 proteins on BLIPO-CUR, pure NK cell membrane fragments and NK cells.

**
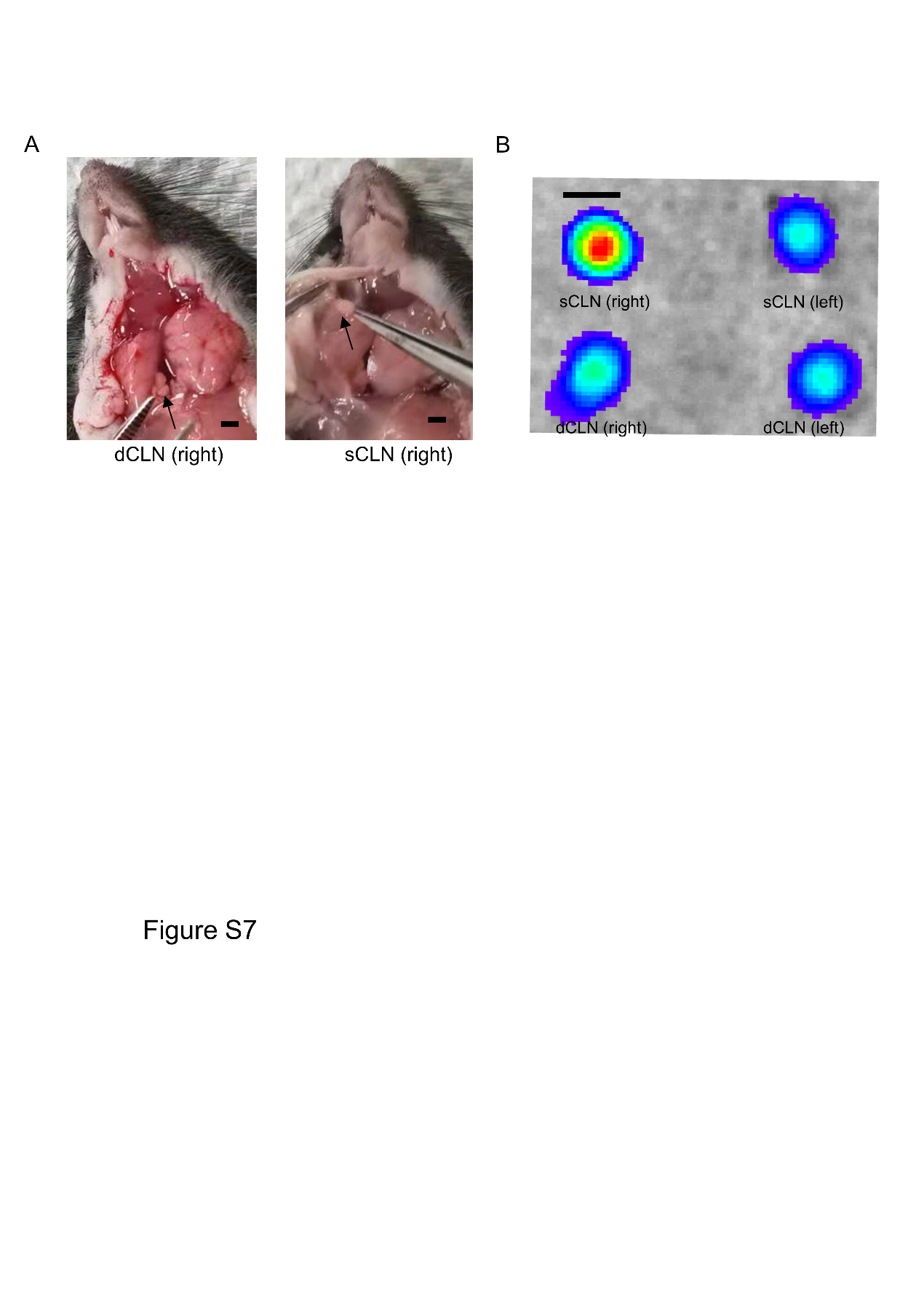
**

Figure S10. The surgically isolated CLNs. sCLN, superficial cervical node; dCLN, deep superficial cervical node; Scar bar is 1 mm.


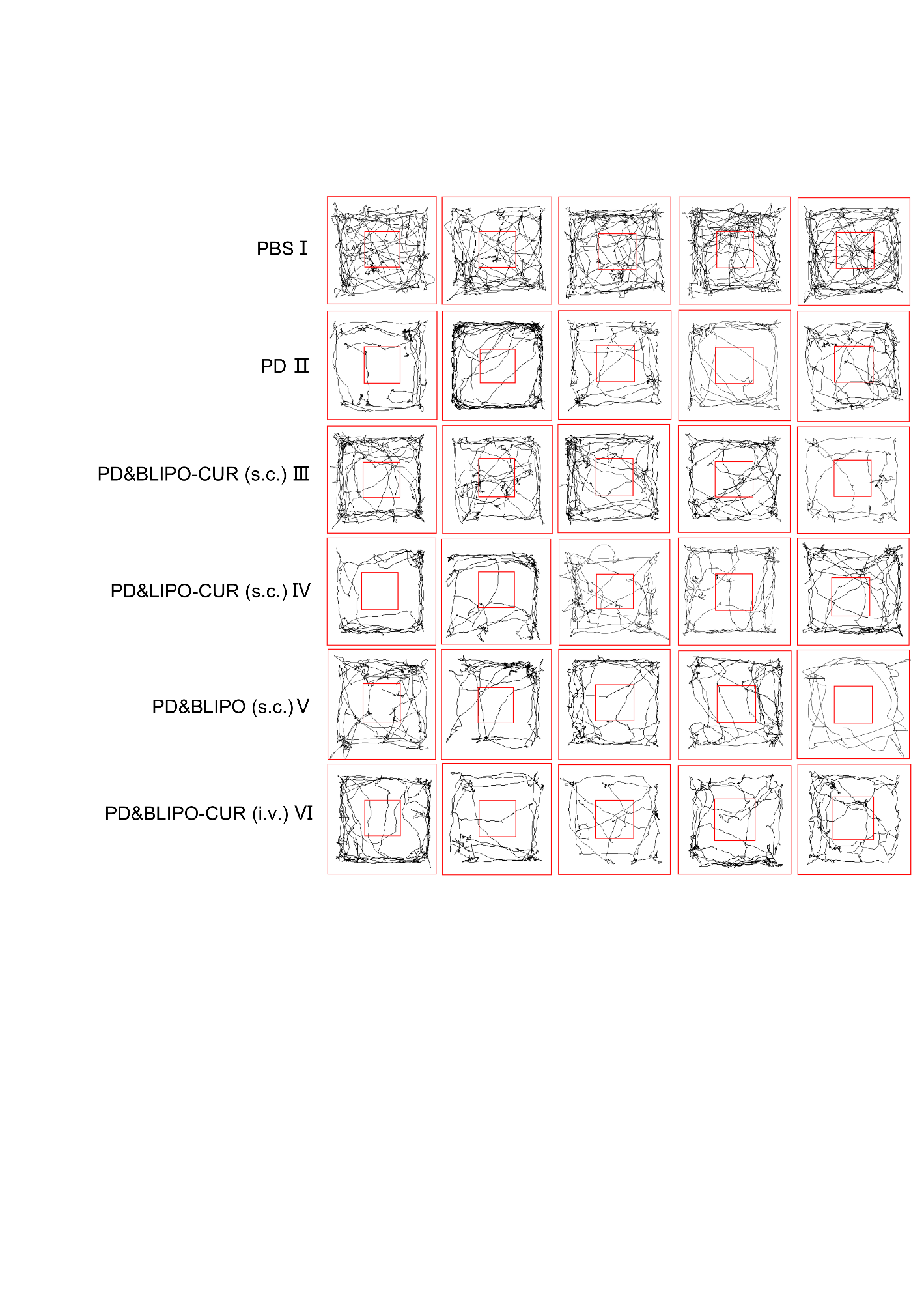


Figure S11. Open-field test. The open filed movement track of the mice from different groups.


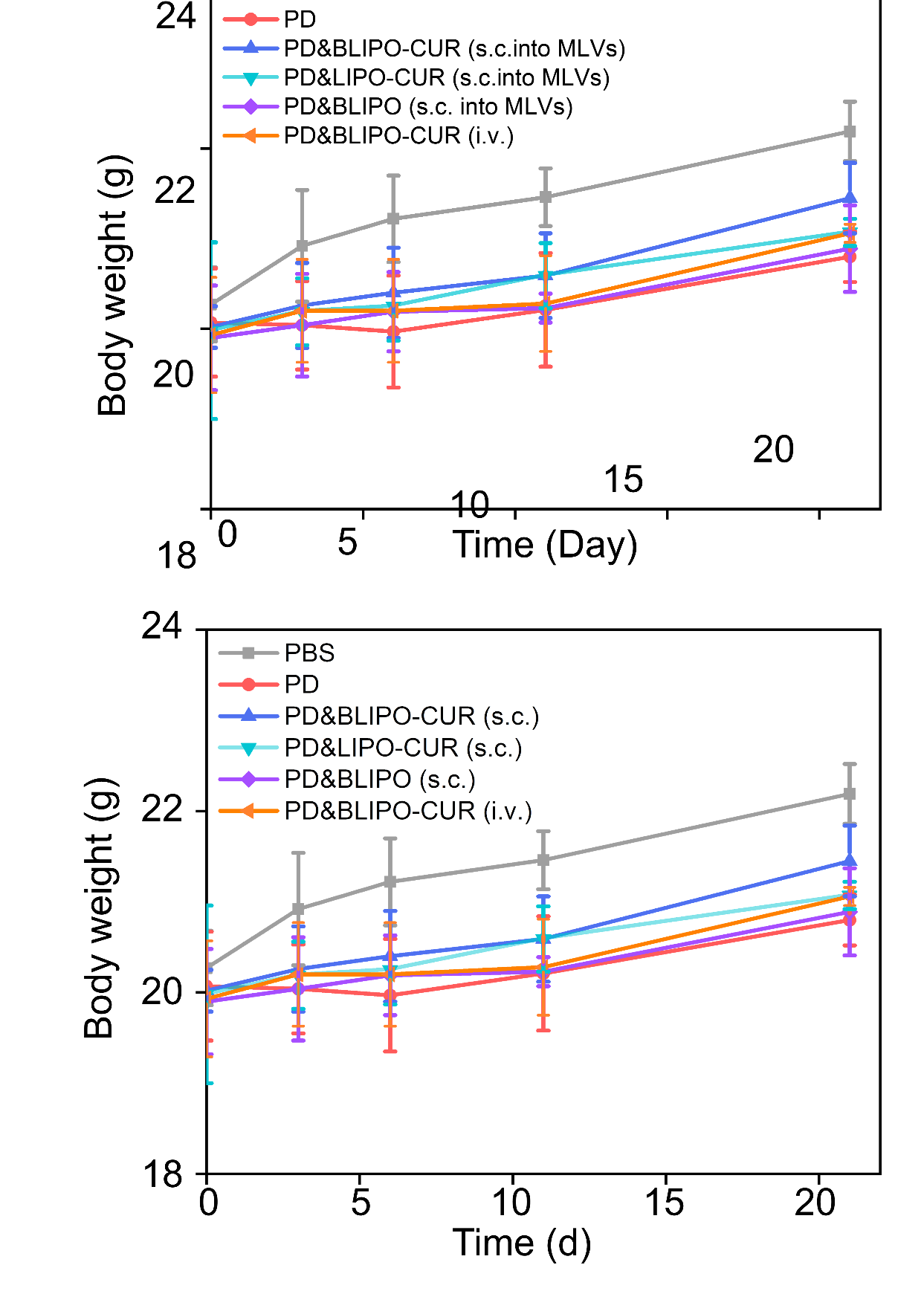


Figure S12. Body weight of mice in all groups. Body weight of mice in all groups were evaluated during the whole experiment. n = 6 animals for each group. All the data represent mean ± S.D.

**
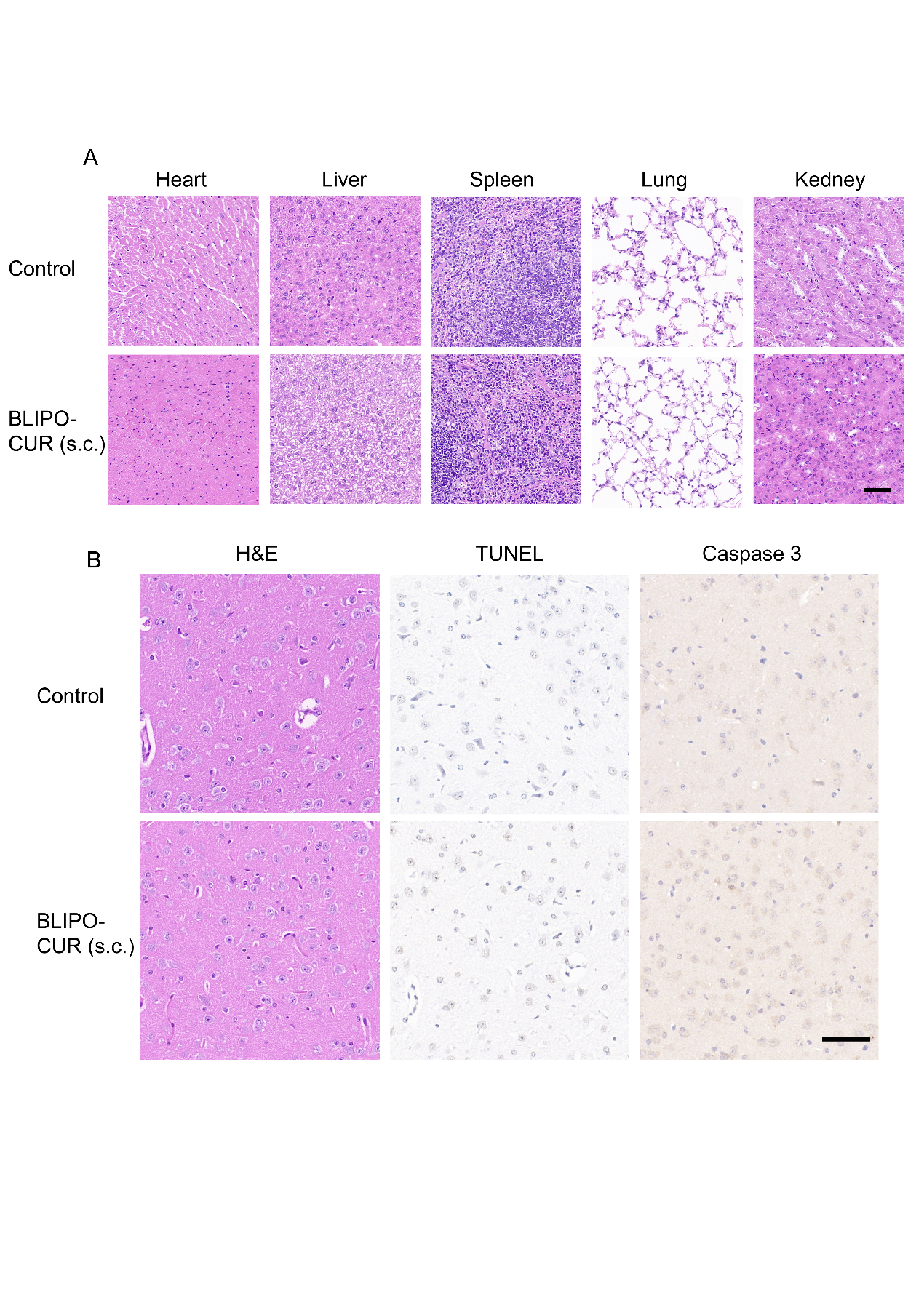
**

Figure S13. Biocompatibility evaluation of BLIPO-CUR. (A) H&E staining of main organs of the mouse treated by BLIPO-CUR. (B) H&E staining of the brain of the mouse treated by BLIPO-CUR. Scar bar, 50 μm.

Table S1. Information of as-prepared BLIPO-CUR


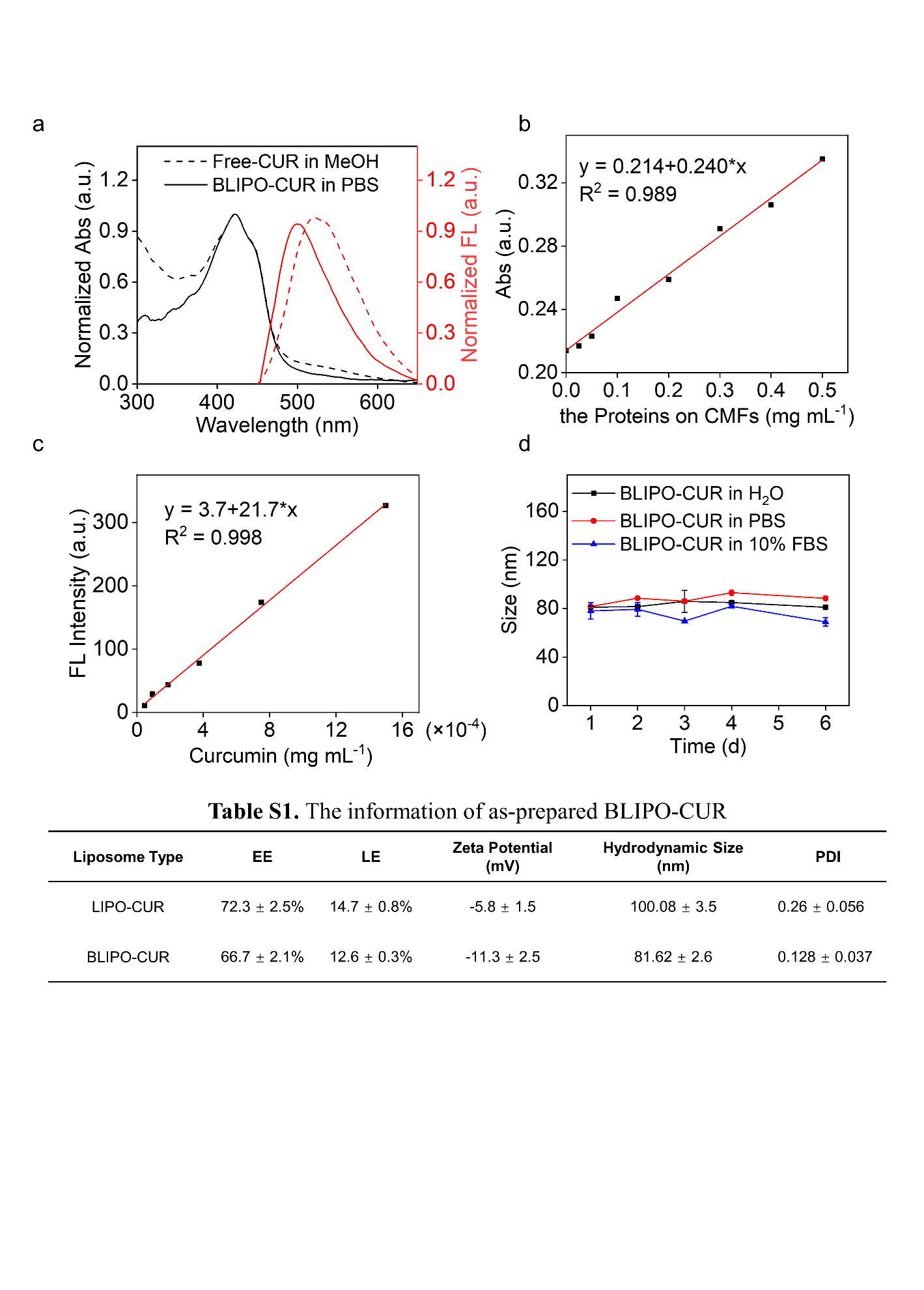


EE: Encapsulation efficiency; LE: Loading efficiency; PDI: Polydispersity index
